# Amniotic membrane promotes doxorubicin potency by suppressing SH-SY5Y neuroblastoma cell angiogenesis

**DOI:** 10.1186/s12885-025-14442-z

**Published:** 2025-06-19

**Authors:** Ahmed M. Abou-Shanab, Shaimaa Shouman, Alaa E. Hussein, Ola A. Gaser, Shireen Magdy, Eman Ashraf, Radwa Ayman Salah, Omaima Idris, Nagwa El-Badri

**Affiliations:** 1 Center of Excellence for Stem Cells and Regenerative Medicine, Zewail City of Science and Technology, Giza, 12578 Egypt; 2 Biomedical Sciences Program, University of Science and Technology, Zewail City of Science and Technology, Giza, 12578 Egypt; 3 Department of Obstetrics and Gynecology, Kasr Al-Ainy University Hospital, Cairo University, Cairo, Egypt

**Keywords:** Neuroblastoma, Amniotic membrane extract, Doxorubicin, NB angiogenesis, Mitochondrial metabolism

## Abstract

**Background:**

Doxorubicin (DOX) remains a mainstay for neuroblastoma (NB) treatment, but side effects hamper efficacy. We previously showed that DOX induces SH-SY5Y NB cell angiogenesis via the PHD-2/HIF-1α axis. Adjuvant therapies offer a promising avenue to improved outcomes. Human amniotic membrane (hAM) extract (hAME) consists of various proteins that exhibit anti-cancer and anti-angiogenic properties. This study investigates hAME as a potential adjuvant for targeting NB angiogenesis when combined with DOX.

**Methods:**

We used cellular, molecular, and biochemical assays to evaluate the antitumorigenic activities of hAME + DOX (D + E) treatment across key hallmarks of SH-SY5Y NB progression: proliferation, cell cycle, angiogenesis, invasiveness, differentiation, and cellular bioenergetics.

**Results:**

D + E treatment significantly suppressed SH-SY5Y cell proliferation, induced cell cycle perturbations, and reduced viability, while protecting bone marrow stem cells and human skin fibroblast normal cells. D + E treatment also countered SH-SY5Y cell invasiveness and promoted a favorable mesenchymal-to-epithelial transition (MET). Importantly, D + E treatment modulated the SH-SY5Y cellular respiration, evidenced by halted glycolytic metabolism, potentially influencing a shift towards oxidative phosphorylation and boosted urea cycle progression. Mechanistically, D + E abrogated DOX's pro-angiogenic effects and inhibited SH-SY5Y cells’ neo-vascularization in a chick embryo model.

**Conclusions:**

These findings suggest hAME as a promising adjuvant therapy for NB, potentially offering an effective and safe treatment strategy by targeting multiple hallmarks of NB.

**Graphical Abstract:**

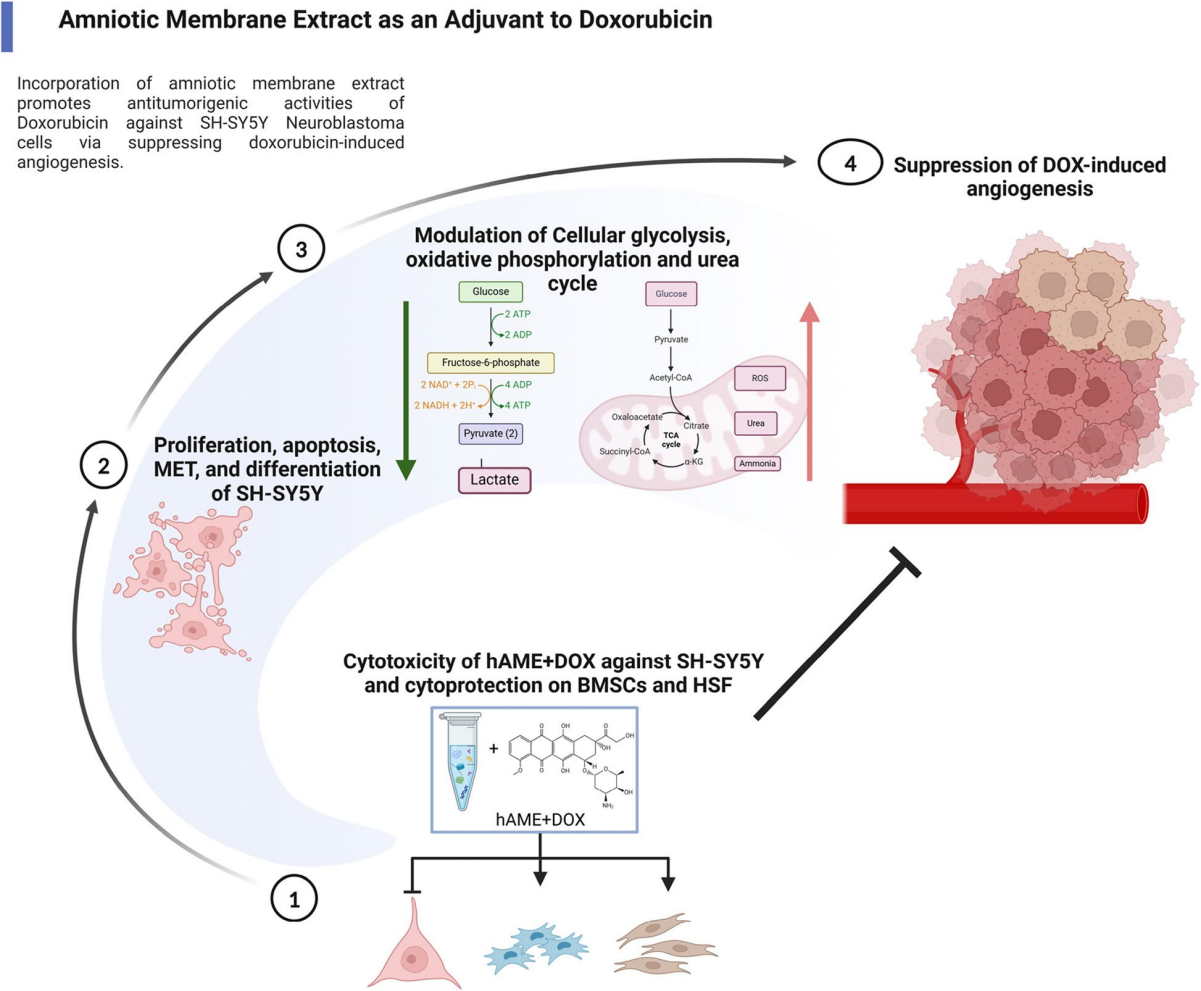

**Supplementary Information:**

The online version contains supplementary material available at 10.1186/s12885-025-14442-z.

## Background

Neuroblastoma (NB) is an embryonic neuroendocrine tumor that originates from a differentiation arrest of sympathoadrenal neural crest cells, promoting their proliferation and oncogenesis [1–4]. It is one of the most common solid malignant cancers in newborns [5, 6]. The majority arises in the adrenal glands, nevertheless, it can occur anywhere along the sympathetic nervous system, including the celiac, paraspinal, and superior cervical ganglia. Infants with low-risk tumors have a survival rate of 88% and regularly report spontaneous regression or differentiation into benign ganglioneuromas [7, 8]. However, older children between 18 months and 12 years have a survival rate of 49%, which deteriorates to less than 10% in young adults (> 12 years) [9–11].

Conventional treatment for NB patients includes tumor resection, radiation therapy, and cytotoxic chemotherapeutics (e.g., DOX) [12, 13], as well as differentiation inducers [14]. A high rate of relapse has been reported after a significant response to chemotherapy [15], presenting an urgent need for the development of adjuvant multi-targeting therapies, especially for high-risk groups. New approaches aim to redirect NB cells from proliferation into differentiation and lineage commitment [16, 17]. More recently, targeting tumor metabolism became a critical aspect in treating cancer, by including agents that hinder the Warburg effect and induce the urea cycle progression [18]. Biologically based approaches could also prove to be valuable in protecting normal cells from the damaging cytotoxic effects of chemotherapeutics [19, 20], especially when using synergistic drug combination therapy.

hAM is a biomimetic material with great promise as adjuvant therapy due to its desirable regenerative capacities and is considered an attractive approach in anticancer therapy [21–23]. The hAM is an avascular membrane surrounding the fetus that consists of two main layers, the chorion and the amnion [24]. After delivery, it is a disposable byproduct and poses little ethical concerns regarding its use in research and therapies [25–27]. Two types of cells reside within the hAM layers, including the amniotic mesenchymal stromal cells (AMSCs) and the amniotic epithelial cells (AECs) [24, 28]. The hAM exhibits anti-inflammatory, hemocompatibility, and anticancer properties [29–34]. The membrane comprises a rich biomatrix and a high content of thrombospondin, plasminogen activator inhibitor, collagen VIII, tissue inhibitors of metalloproteinases, and interleukin-10 and interleukin-1 receptor antagonists [35–40], all of which prevent angiogenesis.

hAME was shown to have anti-cancer effects via controlling the glycolytic and oxidative levels in human HCC-derived Hep3B2.1–7, HepG2, and Huh-7 cells [41]. This was achieved by the hindrance of the Warburg effect and caused an increase in the expression of 6-phosphofructokinase in HepG2 and Huh-7 cells and limited glucose conversion into lactate [41–43]. HAME was found to reduce cell proliferation in prostate cancer (PC3) and downregulate HSP90 gene and protein expression [44]. The hAME was reported to modulate the expression of epithelial-mesenchymal transition (EMT) markers in bladder cancer urothelial cells in a dose-dependent manner while increasing the secretion of TIMP-2. The antitumorigenic activity included downregulating FAK expression, modulating the cytoskeleton actin protein reorganization and small Rho GTPases, and downregulating the bladder cancer Pi3K/Akt/mTOR hallmark signaling pathway [45]. Yet, hAM’s anticancer effects on NB tumors, known for their high angiogenic burden, have not been studied before.

Herein, we used the SH-SY5Y NB cell line as it represents the most frequently cited NB cell line, having been cited over 5000 times in the literature [46]. SK-N-SH represents an I-type cell line used to subclone a few different cell lines in 1978 by Biedler, including SH-SY5Y and SH-EP. Neuroblast-like cells from SK-N-SH were subcloned. The first clone was called SH-SY, which was further subcloned into the SH-SY5, and finally into SH-SH5Y [47]. SH-SH5Y has no amplification of MYCN and no chromosome abnormalities on 1p and 11q, but with gains on 17q [48]. The SH-SY5Y can be differentiated into a more mature neuron-like phenotype with neuroblast-like morphology [49]. We aim to investigate the hypothesis that treating the NB cell line (SH-SY5Y) with hAME combined with DOX results in regression in NB tumorigenic characteristics, including proliferative capacity, metabolism, apoptosis, and MET. We also investigate the effect of hAME + DOX on regulating the neuronal-specific network of the cells. Mechanistically, we unveil the suppression of the pro-angiogenic effect of doxorubicin.

## Material and methods

### hAM collection and hAME preparation

Human placentas were handled as described previously [50]. hAM was obtained from disposed tissues after elective cesarean sections from healthy subjects who were screened negative for blood-borne infections. We obtained written informed consent from all subjects involved in the study, and all study procedures were approved and conducted according to the guidance of the Institutional Review Board (IRB) at Sheikh Zayed Specialized Hospital. All methods were carried out according to relevant guidelines and regulations.

The hAM was gently separated from the chorion membrane and placed on ice, then washed with phosphate buffer saline (PBS, PAN Biotech) with 2% antibiotic/antimycotic. The viability of the hAM cells was confirmed using Calcein-AM fluorescence staining (Molecular Probes, USA) and Hoechst 33,342 (Molecular Probes), then imaged using a Leica DMi8 inverted fluorescent microscope (Leica Microsystems, Wetzlar, Germany) [51].

The hAME was prepared following the Mamedo et al. protocol [52, 53]. hAM was minced and subjected to homogenization and sonication on ice, followed by centrifugation at 17,000 xg for 20 min at 3° C. The supernatant was collected, quantified using the Bradford assay for measuring protein extract concentration, and stored at -80 °C until further use [45, 54].

### Cell culture

SH-SY5Y NB cell line (a gift from the Department of Pathology, Children’s Cancer Hospital 57,357, Egypt) was maintained in RPMI 1640 medium (PAN Biotech, Germany) and DMEM/F12 (Gibco, MA, USA) 1:1 supplemented with 10% heat-inactivated fetal bovine serum (FBS) (BioWest, USA), 1% non-essential amino acids (Lonza, Switerland), 1% streptomycin/penicillin (PAN Biotech, Germany) and 1% L-glutamine (PAN Biotech, Germany) at 37˚ C in a humidified 5% CO_2_ incubator. Human skin fibroblasts (HSF) (ATCC, Manassas, VA, USA) were maintained in DMEM (PAN Biotech, Germany) supplemented with 10% FBS and 1% streptomycin/penicillin at 37˚C in a humidified 5% CO_2_ incubator [55]. Human bone marrow-derived mesenchymal stem cells (BM-MSCs) (ATCC, Manassas, VA, USA) were maintained in DMEM supplemented with 10% FBS and 1% streptomycin/penicillin at 37˚C in a humidified 5% CO_2_ incubator as reported previously [50].

### Evaluation of the metabolic activity of SH-SY5Y, BM-MSCs, and HSF cells

The metabolic activity of SH-SY5Y, HSF, and BM-MSCs was tested upon treatment with hAME and DOX (Sigma Aldrich) compared to the control (Ctrl) group (culture medium without treatment). SH-SY5Y cells were seeded at a concentration of 5000 cells/well and treated with hAME for 4 days at a concentration of 0.5, 1, and 2 mg/ml. SH-SY5Y cells were treated with 0.1 and 1 µg/ml of DOX.

Then, we aimed to investigate whether DOX treatment for 1 day and 4 days was different when combined with hAME at a concentration of 1 mg/ml on SH-SY5Y cells. Thus, we treated SH-SY5Y cells with hAME (1 mg/ml, for 4 days) combined with either DOX (1 µg/ml, for 1 day) or DOX (1 µg/ml, for 4 days) versus Ctrl cells.

SH-SY5Y, HSF, and BM-MSCs were seeded at a concentration of 10,000 cells/well in 96 well plates and treated with hAME (1 mg/ml for 4 days), DOX (1 µg/ml for 1 day), and hAME combined with DOX (D + E) prepared from hAME (1 mg/ml for 4 days) plus DOX (1 µg/ml for 1 day).

Following the treatment, the MTT reagent 3-(4,5-dimethylthiazol-2-yl)-2,5-diphenyltetrazolium bromide (Life Technologies, USA) was added to each well at a final concentration of 5 mg/ml, and incubated for 3 h in a 5% CO_2_ humidified incubator at 37˚C. The formed formazan salts were dissolved in anhydrous dimethyl sulfoxide (DMSO) for 15 min on a shaking plate. The optical density (OD) was quantified at 570 nm with reference to 630 nm using a FLUOstar Omega-microplate reader (BMG Labtech, Cary, NC, USA) [56, 57].

### Cell cycle analysis

The cell cycle analysis was carried out as described [58]. Briefly, after the treatments, SH-SY5Y cells were detached, pelleted, then washed with PBS, and the cell pellet was resuspended for fixation in 5 mL 75% ethanol and stored at -20 °C for 7 days. For analysis, cells were washed twice with PBS, resuspended in 50 µl ribonuclease A enzyme (Qiagen, Germany) at a concentration of 100 µg/mL at 4 °C for 15 min followed by adding 200 µl propidium iodide (PI) to reach a final concentration of 50 µg/mL and incubating at 4 °C for additional 60 min. Cells were then resuspended in 0.5 mL FACS buffer and analyzed for cell cycle progression (10,000 cells/sample) using FACSCalibur (Becton Dickinson) following standard protocols using CellQuest Pro Software (Becton Dickinson). Data analysis was performed using FlowJo v. 10.2 software (Treestar, Ashland, OR, USA).

### Quantitative reverse transcription-polymerase chain reaction (qRT-PCR) assay

For total RNA extraction, TRI Reagent (Sigma-Aldrich, USA) was used according to the manufacturer’s instructions to extract the total cell RNA from SH-SY5Y cells of the different groups: Ctrl, hAME, DOX, and D + E. The concentration and purity of the isolated RNA were determined spectrophotometrically at 260/280 nm using a NanoDropTM 2000/2000c spectrophotometer (Thermo Scientific, Waltham, MA, USA). For cDNA synthesis, 0.25 μg of total RNA was reverse transcribed using a commercially available kit (RevertAid First Strand cDNA Synthesis Kit, Thermo Fisher) before qPCR analysis. The reaction profile using random hexamer primers included incubation at 25° C for 5 min and reverse transcription at 42° C for 60 min. The reaction was stopped at 70° C for 5 min, and the obtained cDNA samples were preserved at − 80° C. Quantitative PCR analysis was performed in triplicate per cDNA sample using HERA PLUS SYBR® Green kit (Willowfort, UK) according to the manufacturer’s instructions. The reaction was carried out in a real-time PCR system (CFX96 Touch™ Real-Time PCR Detection System). Relative gene expression was performed using the delta-delta Ct (-∆∆Ct) method and normalized to the reference gene, β-actin (housekeeping gene). The following PCR cycling parameters were used: initial denaturation at 95° C for 2 min, followed by 40 repeated cycles of 10 s at 95° C, 30–60 s at 60° C, and a final extension at 72° C for 10 min. The primers used are listed in (Supplementary File 2: Table 1).

### Whole-cell protein extraction, SDS–polyacrylamide gel electrophoresis (SDS-PAGE) and Western blot

**Fig. 1:**
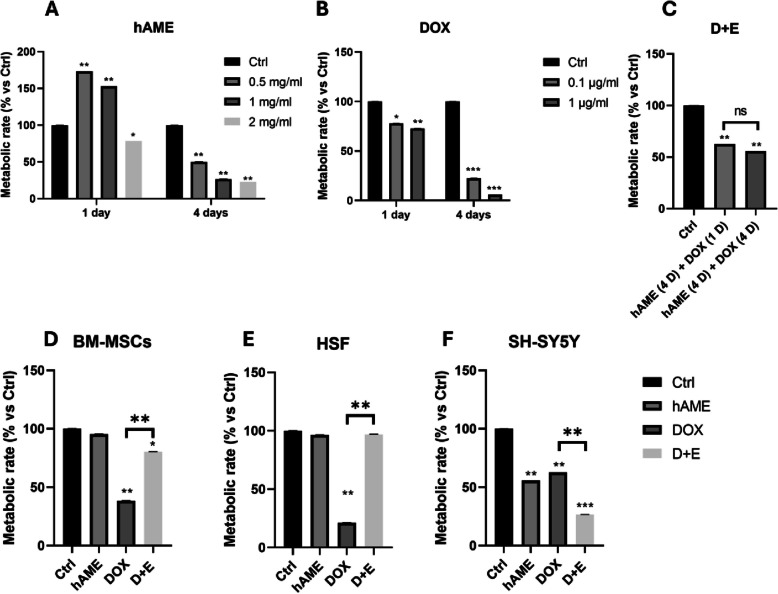
Assessment of SH-SY5Y, BM-MSCs, and HSF cells metabolic activity. **A** SH-SY5Y treated with hAME for 4 days. **B** SH-SY5Y cells treated with DOX. **C** MTT assay for different combinations of hAME and DOX for SH-SY5Y cells. **D** BM-MSCs **E**. HSF cells **F**. SH-SY5Y cells metabolic activity upon hAME, DOX, and D + E treatment

**Fig. 2:**
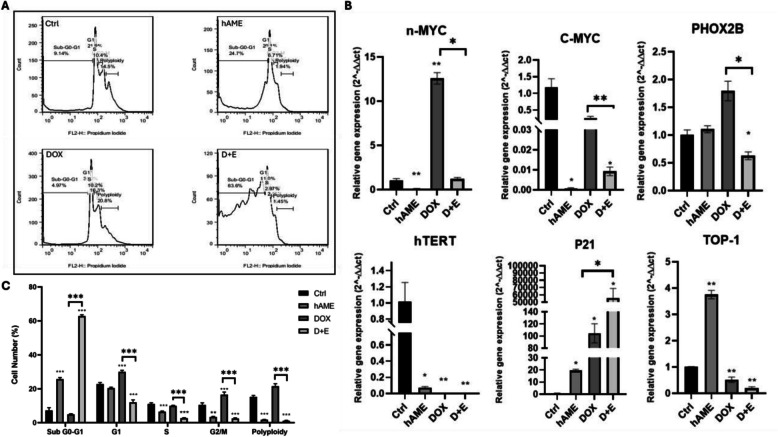
D + E induces G2/M cell cycle arrest in SH-SY5Y cells as well as their proliferation markers. **A** and **C** Cell cycle analysis of SH-SY5Y in Ctrl, hAME, DOX, and D + E groups. **B** Genotypic expression of proliferation markers

**Fig. 3:**
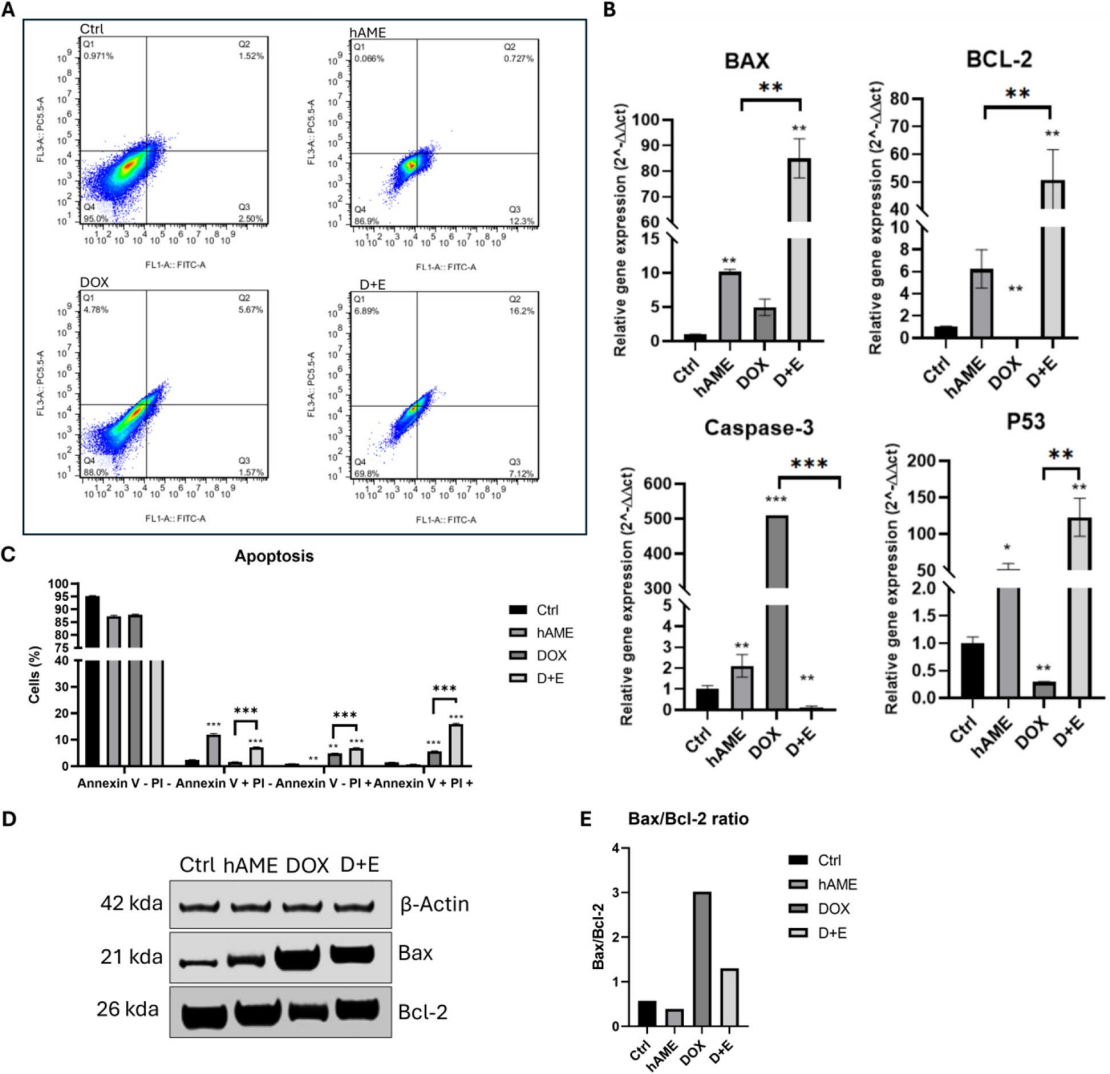
Apoptosis induction in D + E-treated SH-SY5Y cells. **A** Flow cytometry analysis of SH-SY5Y staining by Annexin V and PI. **B** Assessment of apoptotic markers BAX, BCL-2, Caspase-3, and P53 genotypic expression. **C** A graphical percentage of the apoptotic and necrotic populations of both treated and untreated SH-SY5Y cells. **D** Western blot analysis of Bax and Bcl-2 protein expression. **E** Bax/Bcl-2 ratio

Cells were lysed in ice-cold lysis buffer (150 mM NaCl, 50 mM Tris HCl, and 1% Triton X-100, at pH = 8) after adding a protease inhibitor cocktail (Thermofisher Scientific) to a final concentration of 10%. Total protein quantification was determined using Bradford assay and equal amounts of total protein were boiled at 95˚ C for 5 min with SDS-PAGE sample buffer (25% glycerol, 31.2 ml 0.25 M Tris HCl (pH 6.8), 7.5 ml of 10 ml 10% SDS, 0.3 M DTT, and 30 mg of bromophenol blue for each 100 ml). hAME was separated in 7% SDS-PAGE and stained with Coomassie Blue protein stain (BioRad, USA) to confirm the reproducibility of the extraction methodology. Whole-cell protein was separated in 10% SDS-PAGE and the separated proteins transferred onto a polyvinylidene difluoride (PVDF) membrane (Santa Cruz Biotechnology) as previously described [59]. The membranes were blocked in 5% non-fat milk in PBS for 1 h at room temperature, followed by overnight incubation in a shaking roller at 4 ˚C with primary antibodies to beta-actin (Cell Signaling Technology), vimentin (Biorbyt, UK), Snail and Slug (Abcam, UK), N-Cadherin (Abcam, UK), E-Cadherin (BioLegend, USA), BAX (Abcam, USA), Bcl-2 (Abcam, USA), and Tubulin III (BioLegend, USA). Membranes were then washed three times with PBS-Tween 20 (0.1%) before the incubation with goat anti-mouse IgG (H + L)-HRP conjugate or goat anti-rabbit IgG (H + L)-HRP conjugated secondary antibodies (Bio-Rad, USA) at 1:3000 dilution in 5% non-fat milk for 2 h in a shaking roller at 4 ˚C. After 3 × 10 min washing in PBS-T. Membranes were developed with enhanced chemiluminescence detection reagent, clarity™ Western ECL blotting substrate (Millipore, USA). The membrane protein band signals were visualized using ChemiDoc™ MP Imaging System (Bio-Rad, USA). Uncropped blot images in the Supplementary File. 2 Fig. 1, Fig. 2, and Fig. 3.

### Apoptosis analysis

Induction of cellular apoptosis by the anticancer regimens applied to the SH-SY5Y NB cell line was analyzed by flow cytometry (10,000 cells/sample) using Annexin V FITC and PI apoptosis kit (Miltenyi Biotec Inc., Auburn, CA, USA) as per the manufacturer’s protocol.

### Transwell invasion assay

The cell invasion assay was carried out as mentioned previously [60]. SH-SY5Y cell line was cultured in a 24-well plate Transwell system (ThinCert cell culture insert, 8-µm pore diameter; Greiner, Germany) with a seeding density of 2500 cells/well. The transwell was pre-coated with Matrigel basement membrane matrix (Corning, NY, USA) at a final concentration of 1 mg/ml. Briefly, cells were suspended in 200 µL FBS-free medium and seeded into the upper compartments. 500 µl complete medium was added into the lower compartments, supplemented with 20% FBS, after which the cells were left in the incubator at 37 °C for 48 h. Invasive cells on the lower surface of the filter were fixed in 4% paraformaldehyde and stained with 0.5% crystal violet. Cells on the upper surface of the filter were carefully removed using a cotton swab. Stained cells in nine randomly chosen fields were counted under a Leica DMI8 inverted fluorescent microscope (Leica Microsystems, Wetzlar, Germany), and analyzed using ImageJ software (NIH, USA).

### *In-ovo* chick embryo chorioallantois membrane (CAM) assay

The chick CAM is a highly vascularized extra-embryonic membrane that functions for gas exchange, nutrient exchange, and waste removal for the growing chick embryo. It is beneficial as it can function as an angiogenic treatment screening tool, which bridges the gap between cell-based *in-vitro* studies and *in-vivo* animal experimentation. The CAM assay is an underutilized *in-vivo* angiogenic assay, as it is not subject to issues such as ethical approval and animal sacrifice [61–63].

Newly fertilized pathogen-free Egyptian Fayoumi eggs (Poultry Center, El-Azab, El-Fayoum, Egypt) were incubated in an egg-hatching incubator at 37˚ C for 7 days till the 7.5E stage, at which a window was opened on the stamped area of the egg using a mini drill under sterile conditions. The air sac membrane was removed carefully using sterile forceps, and the vascular structure of the attached CAM was carefully undisturbed. To assess the angiogenic potential of SH-SY5Y cells after hAM treatment, a sterile plastic ring was placed on an avascular area of the CAM between two large vessels using sterile forceps. For preparing the sample, about 800,000 cells/pellet were resuspended in 150 µl of sterile egg white albumin and 50 µl of sterile PBS. The cell suspension was inoculated into the plastic ring that was previously placed on CAM. The egg and the inoculated cells were left for 5 min to settle, the window was closed using sterile stretch film, and the eggs were incubated for 7 days. To isolate the CAM, a small cut was made on the edges of the membrane, which was then lifted using sterile forceps, and the chick embryo was euthanized. On a 6-well plate, the isolated membrane was placed in a well filled with 4% paraformaldehyde (PFA) fixative for 4 h. The fixed membrane was stored in PBS at 4 °C, and the newly formed vessels were counted using ImageJ software (NIH, USA).

### Enzyme-linked immunosorbent assay (ELISA)

To quantify the intracellular Lactate dehydrogenase A (LDHA) and vascular endothelial growth factor A (VEGF-A) in SH-SY5Y cells upon treatment with hAME, DOX, or D + E, cells were harvested and lysed. The cell lysates were quantified using Bradford reagent (Sigma-Aldrich, USA). LDHA concentration was quantified in the cell extract from different groups using a commercial LDH kit (BIOSYSTEMS, Spain) following the manufacturer's protocol.

A commercial VEGF-A ELSA kit (Elabscience, USA, CAT: E-EL-H0111) was used according to the manufacturer's protocol. Briefly, samples or standards were added to the wells of the pre-coated ELISA plate with an antibody targeting human VEGF-A, and avidin-horseradish peroxidase (HRP) conjugate. The plate was then incubated and washed for 15 min, after which a substrate was added to initiate the reaction, followed by a blocking solution to stop the reaction. Finally, the optical density was measured at 450 nm using a BMG Labtech FLUOstar Omega plate reader (Germany).

### Biochemical analyses

The quantity of urea, ammonia, lactate, glucose, and alkaline phosphatase in the conditioned medium of experimental and control groups was measured using the following chemicals respectively: commercial urease enzymatic colorimetric assay kit (Diamond Diagnostic, Cairo, Egypt), commercial ammonia kinetic enzymatic with glutamate dehydrogenase kit (SPECTRUM, Cairo, Egypt), commercial lactate plus-liquizyme enzymatic colorimetric assay kit (catalog number 274–001, Spectrum Diagnostics, Cairo, Egypt), commercial glucose enzymatic colorimetric assay kit (Biodiagnostic, Cairo, Egypt) and commercial alkaline phosphatase colorimetric method (Biodiagnostics, catalog number AP1020). The calorimetric method was performed according to the manufacturer’s protocol and the readings were obtained by measuring the OD at 578 nm for urea, 546 nm for lactate, 510 nm for glucose, and alkaline phosphatase using a FLUOstar Omega-microplate reader (BMG Labtech, Cary, NC, USA). Ammonia was quantified at 340 nm using a BTS 350 biochemistry analyzer (BIOSYSTEMS, Spain). Intracellular pyruvate quantity was assessed in the treated cells’ extract using a commercial pyruvate (pyruvic acid) quantitative UV kit (GL1320UPL Diagnostic, Mainz, Germany), and the absorbance was measured at an OD of 340 nm using a FLUOstar Omega-microplate reader.

### Assessment of mitochondrial temperature using Mito Thermo Yellow (MTY) dye

A stock solution of MTY was gifted from Dr. Young-Tae Chang lab [64]. A quantity of 1 mg of lyophilized MTY stored at 4° C was suspended in 100 µl DMSO to make a 10 mM stock solution, stored at -20° C. MTY is light-sensitive and therefore all the following steps were done under dark conditions [65–67]. MTY dye was prepared at a final concentration of 100 nM in DMSO, then further diluted in PBS to a final concentration of 500 nM. Finally, the dye-containing PBS was incubated for 15 min in a 37° C water bath. MTY and MTG (Mito Tracker Green FM, Thermo Fisher Scientific, USA) were prepared as previously reported [66, 67]. Cells were collected and pelleted to measure the mitochondrial temperature of Ctrl, hAME, DOX, and D + E treated SH-SY5Y groups, then washed with PBS. PBS containing 500 nM MTY and 100 µM MTG was added to each well, incubated for 30 min at 37° C and 5% CO_2,_ and examined using flow cytometry (FACSCalibur, Becton Dickinson, USA). Data analysis was performed using FlowJo v. 10.2 software (Treestar, USA) with Overton subtraction analysis for the determination of differences in histograms [68].

### Statistical analysis

The experiments were repeated twice, and the samples were run in at least triplicate. Data are presented as the mean ± SD using GraphPad Prism (Version 8). One-way ANOVA, complemented by mean ± standard deviation (SD) calculations, was used to calculate the *P* values: * *P* < 0.05, ** *P* < 0.01, *** *P* < 0.001 were considered statistically significant. Fluorescence intensity was quantified using ImageJ software (NIH).

## Results

### D + E treatment lowers the metabolic activity of SH-SY5Y NB cells, but not BM-MSCs and HSF cells

Upon preparing hAME and confirming its proteins’ intactness, extracts were quantified and saved at -80 °C for further experimentation (Supplementary File 1: Fig. 1).

Based on literature surveys, hAME and DOX concentrations were chosen. Treatment of SH-SY5Y with 2 mg/mL, 1 mg/mL, or 0.5 mg/mL hAME for 4 days resulted in a decreased metabolic activity (Fig. 1A). DOX treatment significantly reduced SH-SY5Y cell metabolism at both concentrations (0.1 µg/mL and 1 µg/mL) and durations (1 and 4 days) tested (Fig. 1B). Based on these findings, 1 mg/mL hAME and 1 µg/mL DOX were chosen for further evaluation.

Further, we investigated if there is a significant effect for a longer duration (4 days) of DOX treatment from a shorter duration (1 day) at a concentration of 1 µg/mL. Interestingly, the timing of DOX treatment (1 µg/mL) with hAME (1 mg/mL for 4 days) did not significantly affect SH-SY5Y cell activity, suggesting a 1-day DOX treatment is sufficient (Fig. 1C).

Interestingly, hAME treatment (1 mg/mL for 4 days) alone did not affect the metabolic activity of normal BM-MSCs or HSF cells, while DOX (1 µg/mL for 1 day) significantly reduced their viability (Fig. 1D, E). Importantly, the combined D + E treatment (hAME “1 mg/mL for 4 days” and DOX “1 µg/mL for 1 day”) provided a protective effect on both BM-MSCs and HSF cells, counteracting DOX-induced cytotoxicity (Fig. 1D, E). However, in SH-SY5Y cells, all treatments involving hAME (1 mg/mL for 4 days), DOX (1 µg/mL for 1 day), and D + E (hAME “1 mg/mL for 4 days” and DOX “1 µg/mL for 1 day”) resulted in decreased metabolic activity compared to the Ctrl group (Fig. 1F).

### D + E treatment arrests cell cycle progression and downregulates the expression of proliferation markers

hAME and D + E treatments induced distinct cell cycle perturbations in SH-SY5Y cells compared to Ctrl (Fig. 2). hAME and D + E treatment caused a significant G0/G1 cell cycle arrest (Fig. 2A). Notably, D + E displayed a marked decrease in the G1, G2/M, S, and polyploidy phase populations compared to Ctrl (Fig. 2C). hAME and D + E treatment showed a remarkable increase in sub-G0/G1 cell population (Fig. 2C). On the other hand, DOX treatment increased the cell population in G1 and G2/M phases (Fig. 2C). This suggests a potential for both hAME and D + E to inhibit cell cycle progression, while DOX may primarily affect mitotic fidelity.

These observed cell cycle alterations likely reflect modulations in gene expression by hAME and D + E treatments (Fig. 2A). hAME treatment significantly downregulated the expression of genes associated with cell proliferation (telomerase reverse transcriptase (hTERT), cyclin A1 (CCNA1), cellular myelocytomatosis (c-MYC), and n-MYC) and concurrently upregulated genes linked to cell cycle arrest (P21). DOX treatment mirrored the downregulation of proliferation markers (hTERT, and topoisomerase 1 (TOP1)) but also exhibited increased expression of P21, while uniquely upregulating CCNA1 (a cell cycle progression marker) and n-MYC. Interestingly, D + E treatment resembled hAME's effects, with a significant upregulation of P21 (cell cycle inhibitor) and downregulation of TOP1, CCNA1, c-MYC, and hTERT. Paired-like homeobox 2B (PHOX2B) promotes NB malignant transformation during terminal neuroblastic differentiation via activating anaplastic lymphoma kinase. Notably, PHOX2B expression remained largely unchanged with hAME or DOX treatment but was significantly downregulated in the D + E group (Fig. 2B).

Furthermore, the combination treatment (D + E) appeared to enhance the suppressive effects on proliferation markers compared to DOX alone (Fig. 2B). D + E treatment resulted in a significant downregulation of c-MYC, n-MYC, PHOX2B, and CCNA1 compared to DOX. Conversely, P21 expression was significantly higher in the D + E group compared to DOX. Notably, there was no significant difference in TOP1 and hTERT expression between the D + E and DOX treatment groups. In conclusion, these findings suggest that hAME and D + E treatments likely exert their anti-proliferative effects on SH-SY5Y cells through distinct mechanisms. Both treatments appear to induce cell cycle arrest, with hAME potentially targeting multiple cell cycle checkpoints and D + E exhibiting a more pronounced cell cycle arrest. Additionally, both treatments modulate gene expression, with hAME downregulating proliferation markers and D + E further enhancing this suppression.

Top relaxation assay for SH-SY5Y cells after treatments was conducted demonstrating that hAME treatment did not alter plasmid migration, indicating no inhibition of topoisomerase activity. In contrast, treatments with DOX and D + E resulted in halted plasmid migration, confirming that topoisomerase activity was inhibited (Supplementary File 1, Fig. 2).

### hAME promotes DOX efficacy inducing apoptosis in SH-SY5Y cells

Flow cytometry analysis revealed distinct apoptotic profiles following hAME, DOX, and D + E treatments (Fig. 3). Compared to Ctrl group (Fig. 3A), hAME treatment resulted in a significant increase in the early apoptotic population (Annexin V + /PI-) (Fig. 3C). Conversely, the D + E group exhibited a significant increase in cells undergoing late apoptosis (Annexin V + /PI +) compared to both Ctrl and DOX groups (Fig. 3C), suggesting an enhanced effect between hAME and DOX in inducing late apoptosis (Fig. 3A). Interestingly, hAME treatment decreased necrotic cell death (Annexin V-/PI +), while both DOX and D + E treatments displayed an increase in necrotic cells (Fig. 3C).

These observed changes in cell death pathways correlated with modulations in gene expression (Fig. 3B). hAME treatment significantly upregulated the pro-apoptotic gene P53, along with Bax (apoptosis regulator), Bcl-2 (apoptosis regulator), and caspase-3 (apoptosis inducer). DOX treatment primarily induced caspase-3 upregulation without affecting Bcl-2 or P53 expression, while Bax levels remained unchanged. Interestingly, D + E treatment mirrored hAME's effects on Bax, Bcl-2, and P53, all showing significant upregulation. However, D + E treatment uniquely downregulated caspase-3 expression compared to hAME (Fig. 3B).

Analysis of Bax, a pro-apoptotic protein, expression showed an upregulation under all treatment conditions (Fig. 3D). Bcl-2, an anti-apoptotic protein, expression was upregulated in hAME and D + E treatment groups while decreased in DOX group (Fig. 3D). Bax/Bcl-2 ratio is an important indicator of cellular homeostasis and apoptosis regulation; a low Bax/Bcl-2 ratio marks surviving cells while a high Bax/Bcl-2 ratio mark apoptotic cells. In our analysis, the Bax/Bcl-2 ratio decreased in the hAME group while increased under DOX and D + E experimental groups (Fig. 3E), indicating the observed increase in apoptotic cell population in Annexin V/PI staining (Fig. 3A).

### D + E treatment induces MET and decreases SH-SY5Y NB cell invasion

**Fig. 4:**
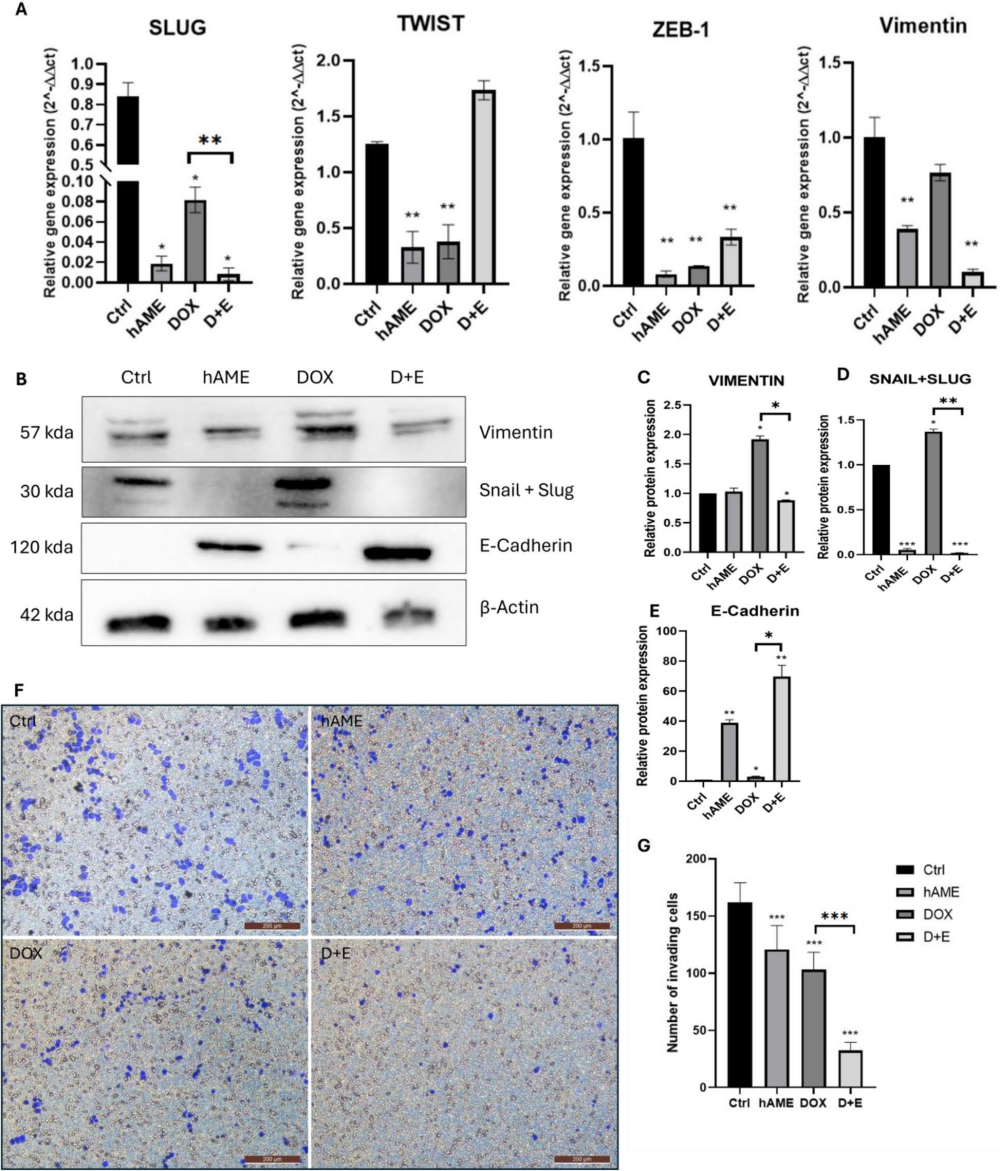
D + E induces MET and halts the invasion potential of SH-SY5Y cells. **A** Genotypic expression of mesenchymal and epithelial markers Slug, Twist, Vimentin, and ZEB-1. **B** Western blotting of MET markers. Relative protein expression analysis of **C**. Vimentin **D** Snail/Slug **E**. E-Cadherin. Analysis of the invasion capacity of the SH-SY5Y cells. **F** Invasion potential of SH-SY5Y groups: Ctrl, hAME, DOX, and D + E. **G** Number of invasive SH-SY5Y cells

hAME and D + E treatments downregulated genes associated with EMT, while DOX exhibited mixed effects (Fig. 4A). TWIST, a transcription factor: TF protein that promotes cancer cell migration and metastasis, expression was significantly reduced in both hAME and DOX groups but remained unchanged with D + E treatment (Fig. 4A). Similarly, vimentin (modulates tumor migration and invasion) and Slug (regulates snail expression and estrogen signaling) expression were significantly downregulated in the hAME and D + E groups compared to control. In contrast, DOX only decreased Slug expression (Fig. 4A). Zinc finger E-box-binding homeobox 1 (ZEB1), a transcription factor promoting EMT, was also significantly downregulated in all treatment groups.

Western blot analysis confirmed the regulation of MET markers at the protein level (Fig. 4B-F). Vimentin protein expression was significantly decreased in the D + E group but increased in DOX-treated cells, with no change observed in the hAME group (Fig. 4C). Conversely, Snail and Slug protein levels were significantly reduced in both hAME and D + E groups. At the same time, DOX treatment resulted in a significant increase (Fig. 4D). Interestingly, the epithelial marker E-cadherin showed a substantial increase in all treatment groups compared to Ctrl (Fig. 4E).

These findings on MET markers’ expression translated to a phenotypical decrease in the invasive capacity of SH-SY5Y cells (Fig. 4F). Compared to the Ctrl group, hAME, DOX, and D + E treatments all resulted in a significantly reduced number of invasive cells through Matrigel-coated transwells (Fig. 4G).

### D + E treatment regulates SH-SY5Y cellular bioenergetics by hindering the Warburg effect and modulating the urea cycle

#### Inhibition of glycolysis in SH-SY5Y cells

**Fig. 5:**
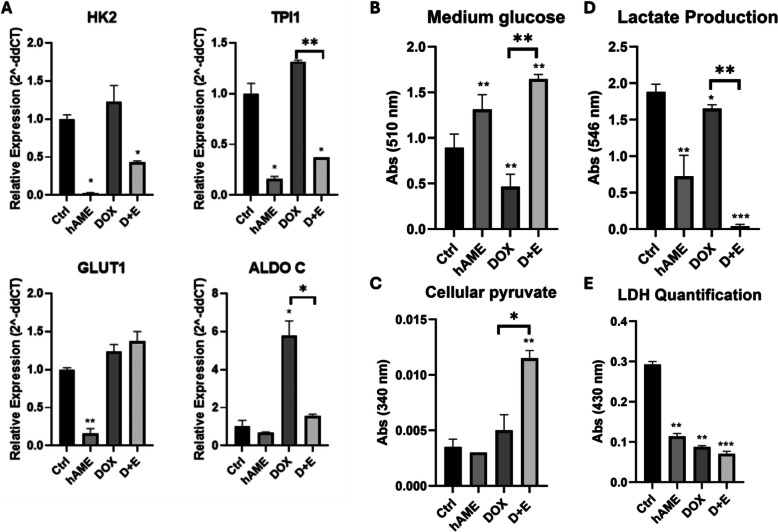
D + E treatment halts SH-SY5Y cellular glycolysis. **A** Genotypic expression analysis of glycolytic genes ALDOC, GLUT1, TPI1, and HK2. **B** Analysis of glucose uptake. **C** Intracellular pyruvate quantification. **D** Lactate production analysis. **E** Intracellular LDHA quantification

The assessment of the Warburg effect in SH-SY5Y cells is of importance as it reflects the impact of D + E treatment on cancer cell metabolism. Both the hAME and D + E groups showed a significant decrease in glucose uptake in comparison to the Ctrl group, as indicated by the quantification of the glucose in the conditioned medium of SH-SY5Y cells (Fig. 5B). On the other hand, DOX-treated cells showed a significant increase in glucose uptake when compared to the Ctrl group as shown by a decrease in conditioned medium glucose (Fig. 5B). The D + E treated group showed a significant reduction in glucose uptake as compared to DOX treated cells (Fig. 5B). SH-SY5Y cellular pyruvate concentration was significantly increased upon treatment with D + E, while no significant change was observed in the DOX and hAME-treated cells (Fig. 5C). As lactate production is considered a waste end-product of glycolysis, our analysis showed a significant decrease in lactate production in hAME, DOX, and D + E groups compared to the Ctrl group (Fig. 5D). Moreover, intracellular LDHA enzyme quantity significantly decreased in hAME, DOX, and D + E as compared Ctrl group (Fig. 5E).

To further assess the blockage of glycolysis in SH-SY5Y cells upon D + E treatment, the genotypic expression of the glycolytic markers triosephosphate isomerase-1 (TPI1) and hexokinase-2 (HK2) were significantly downregulated when compared to the Ctrl group, while the expression of aldolase, fructose-bisphosphate C (ALDOC) and glucose transporter protein type 1 (GLUT1) was not altered. hAME treatment significantly diminished the expression of TPI1, HK2, GLUT1, with no significant change in ALDOC expression. DOX treatment significantly primed the expression of ALDOC and did not alter the expression of HK2, TPI1, and GLUT1 (Fig. 5A).

#### Upregulation of the OXPHOS

**Fig. 6:**
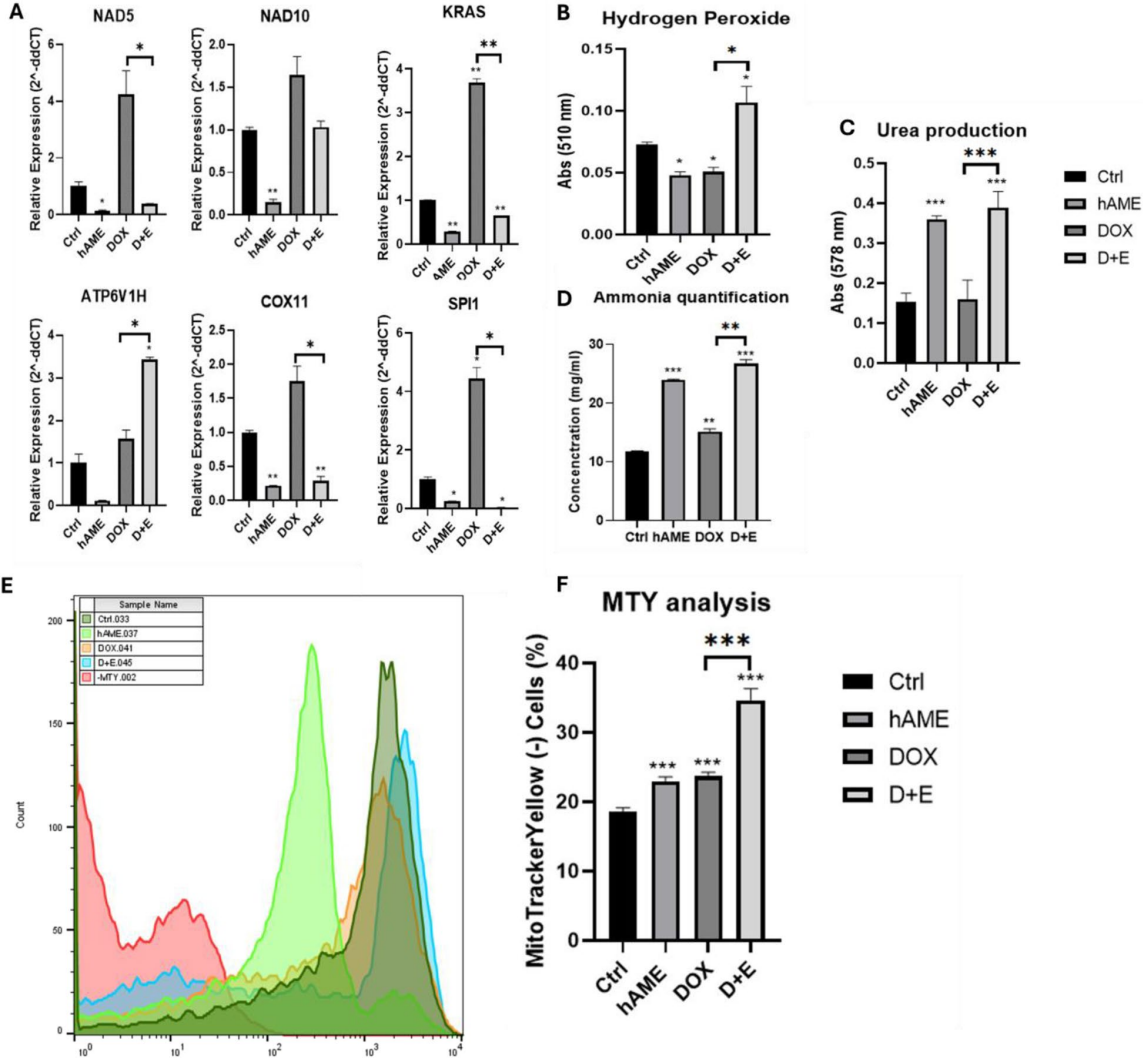
D + E promotes SH-SY5Y mitochondrial metabolism. **A** Genotypic expression analysis of NB biomarkers (KRAS and SPI1) and OXPHOS markers (ATP6V1H, COX11, NAD5, and NAD10). **B** Hydrogen peroxide quantification. **C** Urea production. **D** Ammonia quantification in SH-SY5Y conditioned medium. **E** Flow cytometry analysis of MTY staining. **F** Bar analysis of the intersection between the treated cells and the unstained cell population

Biochemical analysis showed a significant reduction in the production of H_2_O_2_ in the conditioned medium of SH-SY5Y treated with D + E compared to Ctrl (Fig. 6B). To gain further insight into the regulation of OXPHOS, we analyzed the genotypic expression of key enzymes involved in this process. The D + E group showed significant upregulation in the genotypic expression of ATPase H + Transporting V1 Subunit H (ATP6V1H) and a significant downregulation in cytochrome c oxidase copper chaperone (COX11), while the genotypic expression of NADH dehydrogenase subunit 5 (NAD5) and NADH dehydrogenase subunit 10 (NAD10) remained unchanged (Fig. 6A). NAD5, COX11, and NAD10 were significantly decreased in the hAME-treated cells (Fig. 6A). DOX treatment did not alter the genotypic expression of NAD5, COX11, ATP6V1H, and NAD10 (Fig. 6A).

Kirsten rat sarcoma oncogene (KRAS) plays a critical role in controlling NB metabolism by orchestrating multiple metabolic changes. KRAS genotypic expression was significantly downregulated in the hAME and D + E-treated cells, while upregulated in the DOX-treated cells (Fig. 6A). The transcription factor SPI1 plays a prominent role in tumor metabolism and is identified as a biomarker for NB [69]. SPI1 expression was significantly repressed in the hAME and D + E-treated cells, while its expression was not altered in DOX-treated cells (Fig. 6A).

Positively charged MTY dye is used as a fluorescent thermometer to assess mitochondrial heat production by binding to the negatively charged mitochondrial membrane [70]. MTY dye specifically binds to mitochondrial aldehyde dehydrogenase (ALDH2) [71]. Thus, the decrease in MTY incorporation relates to increased mitochondrial temperature. We stained SH-SY5Y cells with MTY and used unstained SH-SY5Y cells as a positive control for conducting OXPHOS. Then, we overlayed flow cytometry histograms and subtracted each treatment group's cell number from the unstained group. As an indication of the activation of OXPHOS in NB cells treated with D + E, the assessment of mitochondrial temperature revealed a significant reduction in MTY dye incorporation, as a reflection of the induction of OXPHOS in these cells (Fig. 6E). Similar results were observed in hAME- and DOX-treated cells. The cells subjected to D + E treatment exhibited a notable MTY dye retention compared to those treated with DOX (Fig. 6F). This observation signifies a higher proportion of cells committed to OXPHOS within the D + E-treated group.

#### Induction of the urea cycle

To study urea cycle modulation in the SH-SY5Y cells upon treatment, we employed biochemical analysis of ammonia and urea concentrations in the culture medium. Our results showed a significant increase in urea in both hAME and D + E groups compared to Ctrl group, while urea concentration in the DOX-treated cells remained unchanged (Fig. 6C). D + E treatment induced a higher urea secretion in the SH-SY5Y cell-conditioned medium when compared to the DOX-treated group (Fig. 6C).

The cells treated with hAME, DOX, and D + E showed a significant increase in ammonia concentration in their conditioned medium as compared to the Ctrl group (Fig. 6D). The D + E treated group showed an increase in ammonia concentration when compared to DOX-treated cells (Fig. 6D).

### hAME combination inhibits doxorubicin-induced angiogenesis in SH-SY5Y

**Fig. 7:**
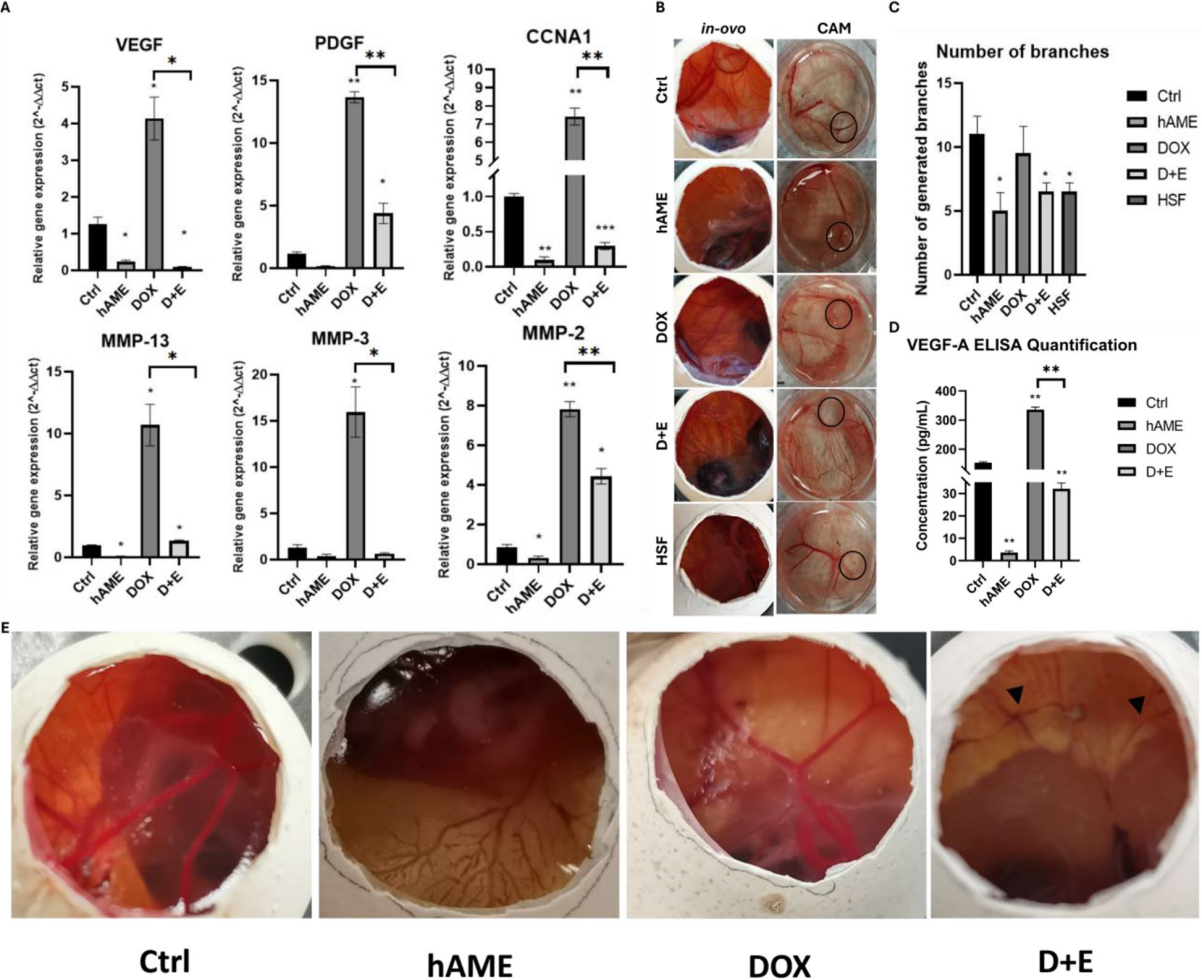
D + E treatment compromised SH-SY5Y neo-angiogenesis. **A** Direct and indirect angiogenic marker genotypic expression. **B** *In-ovo* CAM assay showing inhibited angiogenic stimulatory effect of D + E treated cells. **C** Counting of the number of blood vessel branches generated. **D** ELISA quantification of the VEGF-A protein expression in SH-SY5Y cells. **E** *In-ovo* toxicity assay showing that hAME and D + E but not doxorubicin have anti-angiogenic activity as compared to the Ctrl group treated with PBS

Since angiogenesis is crucial for tumor growth and metastasis, controlling angiogenesis in the tumor environment is of paramount significance for inhibiting cancer progression. Long-term (7 days) direct effect of different treatment conditions on CAM vascular cells showed that both hAME and D + E treatments demonstrated significant toxicity by degrading the vasculature and embryonal death. In contrast, doxorubicin treatment alone did not exhibit any toxicity on CAM vasculature (Fig. 7E).

DOX treatment induced significant upregulation of the mRNA expression of angiogenesis markers platelet-derived growth factor (PDGF), vascular endothelial growth factor (VEGF), Matrix metalloproteinase-2 (MMP-2), CCNA1, MMP-3, and MMP-13 when compared to Ctrl (Fig. 7A) and as addressed previously in [72]. On the contrary, hAME inhibited the expression of VEGF, MMP-2, MMP-13, and CCNA1 genes, with no statistically significant difference in the expression of MMP-3 (Fig. 7A). The D + E-treated group showed significant downregulation of angiogenic markers VEGF, PDGF, MMP-2, MMP-3, MMP-13, and CCNA1 genes when compared to Ctrl.

The protein expression of VEGF was analyzed in the SH-SY5Y cells using ELISA. As shown in Fig. 7D, VEGF was significantly downregulated in hAME and D + E-treated groups, while a significant upregulation in the DOX-treated SH-SY5Y group was observed (Fig. 7D).

SH-SY5Y cell line is known to have strong angiogenic potential, forming sprouting blood vessels due to the secretion of pro-angiogenic factors [73, 74]. We evaluated this potential after treating NB cells with hAME, DOX, and D + E compared to normal HSF cells using *In-ovo* CAM assay. DOX treatment promoted angiogenesis in SH-SY5Y cells (Fig. 7B, C) compared to Ctrl and HSF cells (Fig. 7B, C). Both hAME and D + E-treated SH-SY5Y NB cells (Fig. 7B) showed lower angiogenic potential compared to that of Ctrl and normal HSF cells (Fig. 7C). A comparison between the number of blood vessel branches around the seeded cells was analyzed (Fig. 7C).

### D + E treatment induces SH-SY5Y differentiation into neuronal lineage

Our investigation into SH-SY5Y differentiation revealed distinct effects of hAME, DOX, and D + E treatments (Supplementary File 1, Fig. 3). D + E treatment significantly upregulated the expression of key neuronal markers, including microtubule-associated protein 2 (MAP-2), class III β-tubulin (TUBIII), and neural cell adhesion molecule (NCAM) (Supplementary File 1, Fig. 3). Kruppel-like factor 4 (KLF4), a transcription factor promoting neuronal differentiation, exhibited a similar pattern to the neuronal markers (Supplementary File 1, Fig. 3). D + E treatment showed a significant upregulation of KLF4. Beta-tubulin III protein expression was shown to be upregulated. Interestingly, the cells showed an enhanced ability to form neurospheroids in the D + E group, as the formed neurospheroids were much larger than those of the hAME group. Also, D + E treatment promoted the simultaneous expression of both SOX-2 and OCT3/4 in SH-SY5Y cells. This was accompanied by a significant increase in alkaline phosphatase quantity in the D + E treated groups. Additionally, upon comparing the D + E to the DOX group, the D + E treatment showed a significant increase in alkaline phosphatase (Supplementary File 1, Fig. 3). To confirm that the formed neurospheroids are not characteristics of cancer stem cells (CSCs), we analyzed CSC markers expression. Our data showed significant downregulation of ABCG2, ALDH1, and Nestin under D + E treatment as compared to the Ctrl group (Supplementary File 1, Fig. 4).

## Discussion

Given the potential therapeutic applications of biological agents like hAME, a thorough understanding of their composition and targeted pathways is crucial. Our literature survey has led to the identification of several hAME proteins, based on liquid chromatography-mass spectrometry (LC–MS)-based label-free protein quantitation [75–77], and their proposed associated roles in regulating key biological pathways of NB, including proliferation, apoptosis, EMT, angiogenesis, differentiation, glycolysis, and OXPHOS. (Supplementary File 1: Table 2) provides a detailed summary of the hAME proteins and their respective functions within these pathways.

In our study, D + E treatment significantly decreased metabolic activity in SH-SY5Y cells, but not BM-MSCs and HSF cells, suggesting selective targeting of cancer cells and significantly reduced SH-SY5Y cell viability. These findings align with previous reports demonstrating the antitumorigenic properties of hAM components and culture-conditioned medium against various cancers. A study by Magatti M. et al. showed that AMSCs arrest the cell cycle in G0/G1 phase most probably by downregulating the expression of genes associated with cell cycle progression, including cyclin-dependent kinase (CDK-2, CDK-4, CDK-6) and cyclins (cyclin D2, cyclin E1, cyclin H) while up-regulating p21 and p15 antiproliferative genes [22, 78]. Di Germanio et al. demonstrated that conditioned medium derived from rat amniotic epithelial cells inhibited the proliferation of HepG2 cells, most probably via secreting soluble factors in the medium [51, 79]. It was also reported that hAME arrested HuH-7 and HepG2 cells at the G2/M phase and was accompanied by massive DNA damage [80]. Furthermore, we found that D + E treatment downregulated proliferation markers and induced G0/G1 cell cycle arrest in SH-SY5Y cells. We observed dysregulation in the cell cycle pattern of D + E-treated SH-SY5Y cells, potentially due to chromosomal instability and late apoptosis.

Suppression to EMT is shown by upregulation of E-Cadherin and downregulation of vimentin, snail, and slug expression which may account for the halted invasion potential of SH-SY5Y after D + E treatment. Upregulation in the mesenchymal markers in DOX-treated SH-SY5Y cells (Fig. 4B) in our study corresponds to the previously reported mechanism by which DOX-treated cells escaped senescence by inducing their migration potential and chemotherapy resistance [81–83]. Sequential reprogramming factors are involved in MET induction such as TGF-β, KLF-4, and Slug [84]. In our study, there was an upregulation in the expression of KLF-4 in the D + E treatment group which could be linked to KLF-4 signaling in the induction of the expression of E-cadherin, allowing the reprogrammed cells to acquire epithelial morphology and characteristics [85].

SH-SY5Y cells treated with D + E expressed neuronal differentiation-specific markers, such as TUJ, MAP-2, and NCAM [86, 87], in addition to a significant reduction in genotypic expression of ID-1, -2, and -3, all of which are usually highly expressed in NB cells [88, 89]. In particular, ID1 blocks cell response to growth inhibition by p21 [90]. This downregulation in the expression of IDs and upregulation of p21 upon D + E treatment supports proposing the reprogramming shift of NB cell fate into differentiation. In a recent study, NB cell lines treated with all-trans retinoic acid downregulated the expression of several transcription factors, including n-MYC, GATA3, PHOX2B, and ASCL1 [91]. These findings indicate that treatment with retinoic acid can reprogram the enhancer landscape, resulting in down-regulation of n–MYC expression causing proliferative arrest and sympathetic differentiation [91]. These results are consistent with our reported downregulation in the expression of n-MYC and PHOX2B genes in the D + E treatment group (Fig. 2K). Additionally, the ability of D + E-treated cells to form neurospheroids further functionally confirms the neural lineage specification in SH-SY5Y cells [92]. The simultaneous expression of SOX-2 and OCT3/4 in SH-SY5Y observed in our study further supports their developmental regulation during fate commitment [93–97]. Overexpression of alkaline phosphatase was shown to upregulate the expression of neurogenic differentiation markers in SH-SY5Y [98], which aligns with our data.

The Warburg effect stipulates that mitochondrial dysregulation and subsequent stimulation of glycolysis prime cancer development and may account for cancer resistance to cytotoxic chemotherapeutics [99, 100]. NB and other cancer cells thus undergo the Warburg effect as the primary source of energy generation instead of OXPHOS [101]. In NB, cells acquire enhanced glycolysis and oxidative phosphorylation-associated genotypic and protein expression, as well as the capacity to detoxify reactive oxygen species (ROS) in normoxic conditions [102]. In cancer cells, most of the pyruvate is converted into lactate by suppressing the OXPHOS pathway (mitochondrial pathway). In our study, D + E treatment impaired the glycolytic metabolism in cancer cells as evidenced by the changes in glucose consumption, cellular pyruvate quantity, lactate production, and the expression of glycolysis-related genes. As we showed in Fig. 5, D + E treatment caused a decrease in lactate production, and an increase in intracellular pyruvate, in addition to a reduction of the glycolytic genes TPI1 and ALDOC and glucose consumption. hAME has been reported to play a role in inhibiting cancer glucose metabolism by more than 50% in various cell lines, including PC3, WiDr, PANC-1, HepG2, and Hep3B2.1–7 [103]. This data indicates that cells are dependent on other pathways than glycolysis to secure sufficient ATP molecules. The mode of ATP production relies on the fate of pyruvate, the end product of glycolysis, which fate in turn relies on the enzymes pyruvate dehydrogenase (PDH) and lactate dehydrogenase (LDH) [104]. Also, the inhibitory effect of D + E treatment on KRAS expression as well as lactate production supports glycolysis blockage and shifts into oxidative phosphorylation. This is due to the recent reports that mutant KRAS increases the expression of GLUT1 and rate-limiting glycolytic enzymes, including hexokinases (HK), phosphofructokinase 1 (PFK1), and lactate dehydrogenase A (LDHA), promoting glycolytic activity and increasing lactate production [105, 106].

D + E-treated SH-SY5Y cells upregulated ATP6V1H gene expression, which encodes the subunit H of vacuolar ATPase, an OXPHOS marker, as well as increased their ROS production. The observed metabolic shift suggests enhanced mitochondrial respiration, corroborating established evidence that differentiated cells primarily utilize the OXPHOS pathway for energy production rather than glycolysis [107–109], due to a reduction in ATP demand [110]. This induction in mitochondrial respiration was also demonstrated by an upregulation in the mitochondrial temperature as assessed by MTY dye [68, 111]. Thermogenesis is interconnected with oxidative metabolism, which is regulated primarily by mitochondria. Given the inherent inefficiency of ATP synthesis within the mitochondria, a fraction of respiratory energy is dissipated as heat [112, 113]. Thus, mitochondrial temperature in cancer cells is expected to be lower than that of normal cells [68]. The reduced mitochondrial temperature in cancer cells compared to normal cells was reported by Chrétien et al*.* to be due to defective mitochondrial respiration and primed glycolysis to meet their energy supply requirement [66]. The blockage in glycolysis and upregulation of OXPHOS induced the observed neuronal differentiation of SH-SY5Y cells.

Along with metabolic switching in NB, the anabolic pathways, such as glutaminolysis that provide the building blocks for proteins, lipids, and DNA biosynthesis are altered [114–118]. Also, it was demonstrated that some genes of the urea cycle are either overexpressed or silenced in different cancer types, providing metabolic benefits to tumor survival, proliferation, and growth by modulating the availability of urea cycle-related metabolites, such as ammonia [119]. For instance, ammonia is commonly accumulated in cancer cells, and in contrast to the toxic effect of excess ammonia under normal physiological conditions, cancer cells can use ammonia and recycle it for amino acid and nucleic acid synthesis, aiding tumor proliferation [120, 121]. Our data show that the D + E group primed urea cycle progression, releasing more urea as a waste product, while the ammonia, a by-product of glutaminolysis, was not metabolized in SH-SY5Y cells, but secreted into the conditioned medium (Fig. 6). These data support the modulatory role of D + E treatment on SH-SY5Y cells' respiration and bioenergetics, via blocking glycolysis and hindrance of the Warburg effect. This is of importance for cancer progression, as the induction of OXPHOS due to SH-SY5Y0 differentiation and the progression of the urea cycle blocks ammonia recycling.

Our data show that hAME and D + E treatment reduced the pro-angiogenic effect of DOX (Fig. 7) on chick embryos. The angiogenic stimulatory effect of DOX was reported to compromise its therapeutic outcomes in cancer [122, 123]. DOX exerts its anti-cancer effect via inducing DNA damage, production of free radicals, and inhibition of topoisomerase II [124, 125], but can simultaneously upregulate the expression of the pro-angiogenic factors PDGF and VEGF [123] as well as upregulating normoxic HIF1-alpha [126]. We previously addressed that doxorubicin-induced angiogenesis is modulated via the PHD-2/HIF-1alpha axis [72]. Herein, our data show that hAME treatment reduced the genotypic expression of the pro-angiogenic factors PDGF and VEGF, which were downregulated in both hAME and D + E treatment groups (Fig. 5). hAM-derived epithelial stem cells were reported to produce soluble factors to inhibit MMPs, which participate in the inhibition of both angiogenesis and tumor progression [38]. In our study, the genotypic expressions of MMP-2 and MMP-13 were downregulated upon hAME and D + E treatments. In contrast, hAM is shown to have contradictory pro- and anti-angiogenic potential depending on the physiological conditions, including cancer cells, *in-vitro* systems of proliferating normal cells, and some *in-vivo* models of wound healing [23, 127–131].

While our study provides valuable insights into hAME's potential as an adjuvant to DOX for NB therapy, it is essential to acknowledge certain limitations. Our analyses focused exclusively on one NB cell line (SH-SY5Y), so future research should involve other NB cell lines. More research should be done to identify the differential regulation of MET marker expression under different treatment conditions. Additionally, further optimization of hAME's potency and delivery methods, as well as translation into *in-vivo* preclinical models, are necessary to fully realize its therapeutic potential. Moreover, long-term experiments should be done to evaluate prolonged hAME efficacy and safety. By addressing these future directions, we can contribute to a more comprehensive understanding of hAME's role in cancer treatment.

## Conclusions

In summary, this study explored the potential of hAME as an adjuvant therapy for NB in combination with DOX. D + E treatment enhanced cytotoxicity through increased apoptosis and cell cycle arrest and displayed anti-invasive and anti-angiogenic properties. Notably, D + E treatment promoted the expression of neuronal markers in NB cells, suggesting a potential reprogramming towards a more differentiated state. Furthermore, D + E treatment altered NB mitochondrial function, potentially hindering the Warburg effect and modulating the urea cycle. These findings strongly suggest the therapeutic potential of hAME as an adjuvant to DOX in NB treatment.

## Supplementary Information

Supplementary Material 1.

Supplementary Material 2.

## Data Availability

The data used and/or analyzed during the current study are available from the corresponding author upon reasonable request.

## Abbreviations

NB: Neuroblastoma
hAM: Human amniotic membrane
DOX: Doxorubicin
MET: Mesenchymal to epithelial transition
hAME: Human amniotic membrane extract
EMT: Epithelial-mesenchymal transition
OXPHOS: Oxidative phosphorylation
VEGF-A: vascular endothelial growth factor A
