# Supplementary material for: Amniotic membrane promotes doxorubicin potency by suppressing SH-SY5Y neuroblastoma cell angiogenesis
Source: BMC Cancer. 2025 Jun 19;25:1021. doi: 10.1186/s12885-025-14442-z (PMC12180182; doi:10.1186/s12885-025-14442-z)
Supplement: Supplementary file 1 — Supplementary Material 1. [file 12885_2025_14442_MOESM1_ESM.docx]

**Preparation and confirmation of the hAM intactness and viability**

The freshly collected hAM was examined under the bright field showing AECs (Fig. 1A). hAM sample staining with Calcein-AM fluorescence staining (Fig. 1B) and counter-stained with Hoechst 33342 for nuclear staining (Fig. 1C) confirm the viability of the AECs. Three different hAM samples were homogenized and quantified, followed by running three different extracted hAME on SDS-PAGE to confirm the reproducibility of the extraction method. Fig. 1D. shows the intactness of the protein bands.

**
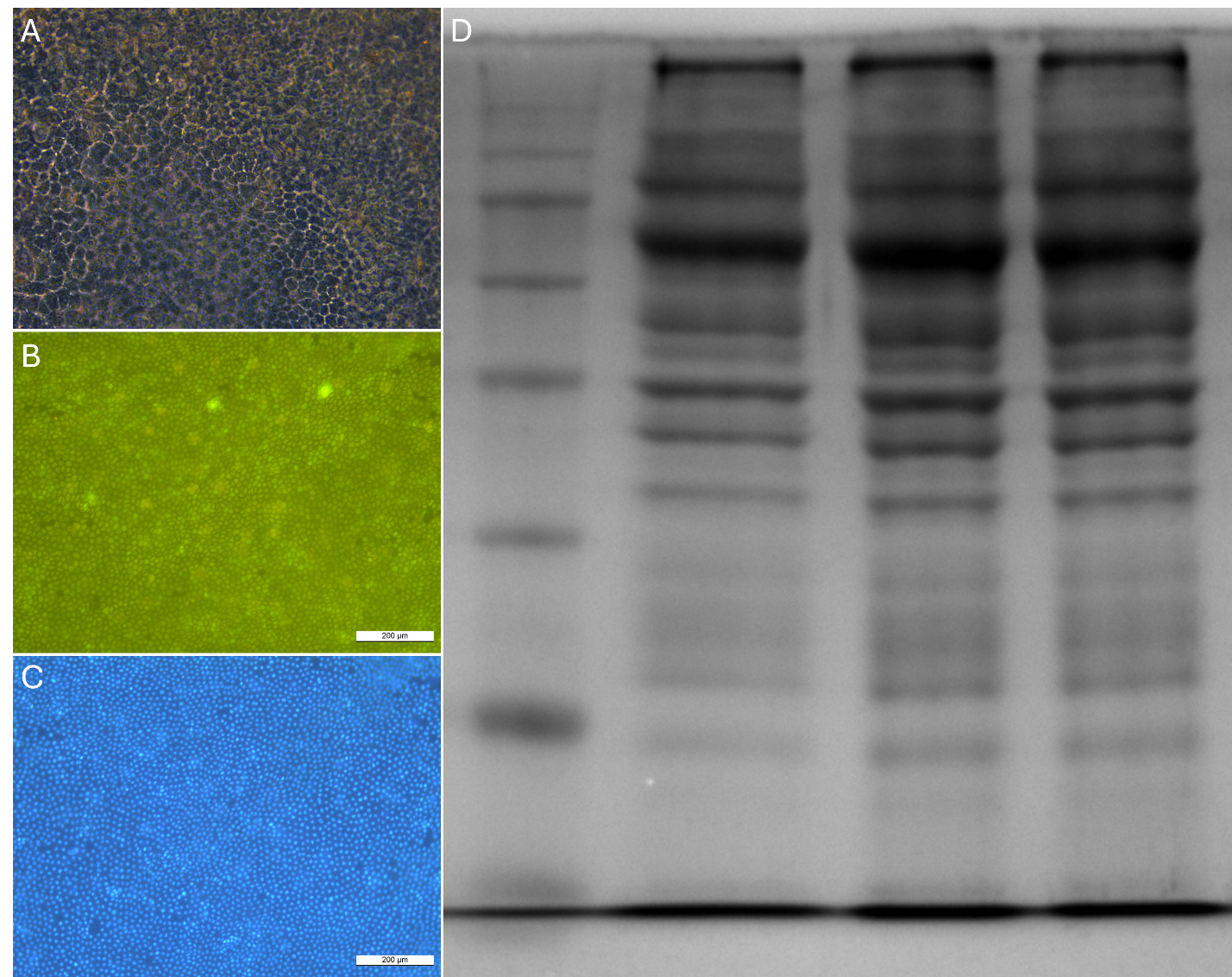
**

**Fig. 1 hAM viability and intactness.** (A) hAM under brightfield microscopy (B) hAM epithelial cells staining with Calcein-AM stain. (C) Nuclear staining of hAM epithelial cells with Hoechst 33342. (D) SDS-PAGE for three different hAME samples.

###
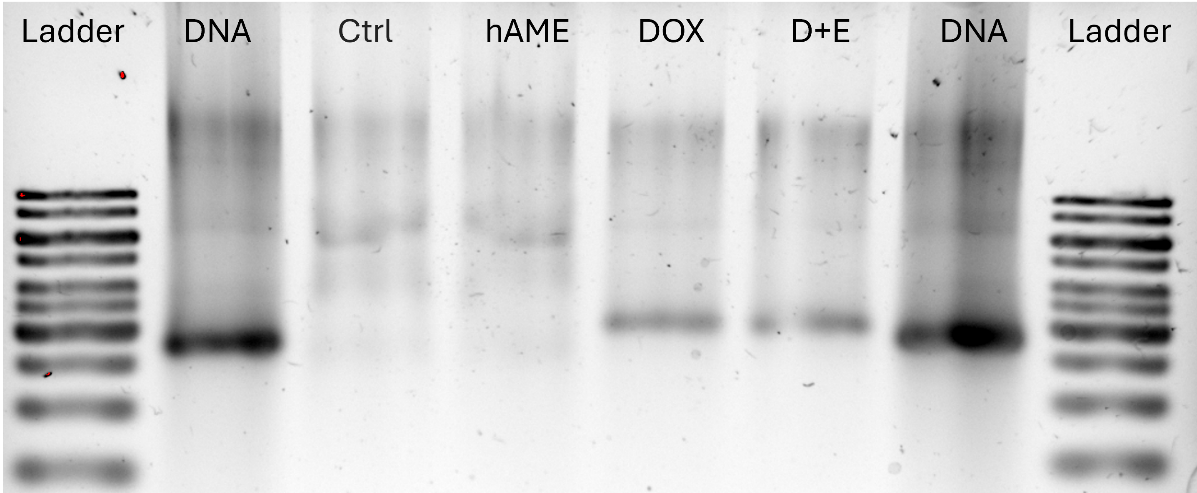


### Fig. 2. Top relaxation assay for SH-SY5Y after treatments demonstrating that hAME treatment did not alter plasmid migration, indicating no inhibition of topoisomerase activity. In contrast, treatments with DOX and D+E resulted in halted plasmid migration, confirming that topoisomerase activity was inhibited, and plasmid DNA remained in its supercoiled form. For this assay, we used whole-cell lysates from treated cells and incubated them with assay buffer containing plasmid DNA.


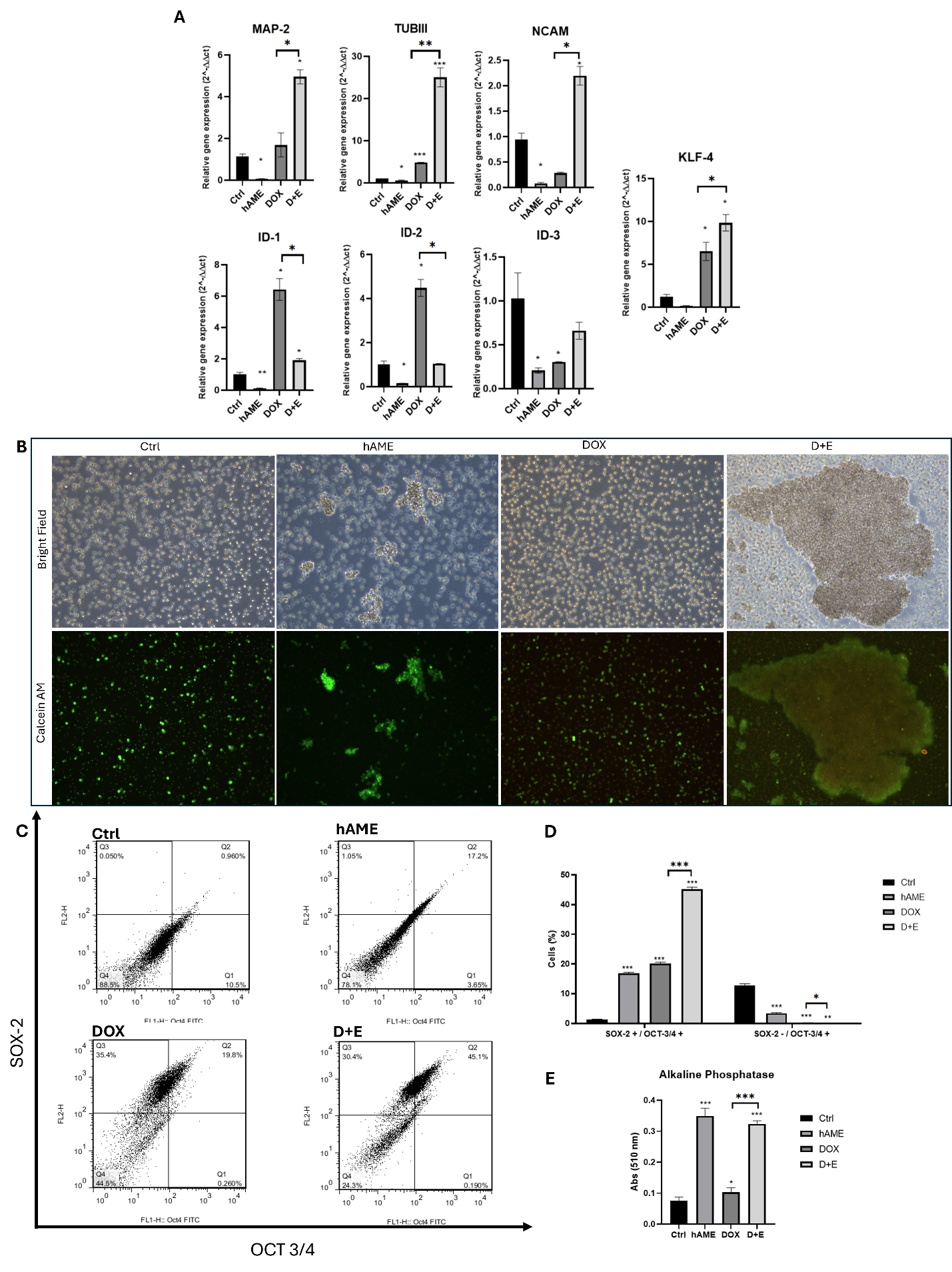


**Fig. 3 D+E treatment induces SH-SY5Y cell differentiation into neuronal lineage.** **A.** Genotypic expressions of the neuronal-specific markers TUJ, NCAM, and MAP-2, KLF-4, and ID-1, -2, and -3. **B.** Neurospheroids formation efficacy in SH-SY5Y cells treated with hAME, DOX, and D+E as well as Ctrl. **C.** Expression of the pluripotency markers OCT3/4 and SOX-2 in Ctrl, hAME, DOX, and D+E treated SH-SY5Y cells. **D.** The percentage of cells expressing OCT3/4 and SOX-2. **E.** Alkaline phosphatase quantification assessment in the conditioned medium of the Ctrl, hAME, DOX, and D+E treated cells.

**Fig. 4 D+E treatment downregulates CSC markers: ABCG2, ALDH1, and Nestin expression in SH-SY5Y NB cells.**

**Table. 1**: hAME proteins and their biological function.

| **Protein** | **Function** | **Ref.** |
| --- | --- | --- |
| **Proliferation** | | |
| Nucleobindin 1 | suppressed cancer cell proliferation | (1) |
| Pentraxin-related protein PTX3 | suppresses the tumor proliferation triggered by DHT or FGFs in prostate cancer cell line TRAMP-C2 and breast cancer cell line S115 | (2-4) |
| Paraoxonase 2 | decreases OC cell proliferation by inhibiting insulin-like growth factor-1 (IGF-1) expression and signaling. | (5) |
| FAS-associated factor 2 | a multifunctional tumor suppressor including death-promotion (both apoptosis and necrosis), cell cycle arrest, anti-inflammation, and anti-angiogenesis | (6) |
| CD109 antigen (TGF-beta-1-binding protein) | Reduced the proliferation of epithelial ovarian cancer cells and caused G0/G1 cancer cell cycle arrest. | (7) |
| CD9 antigen | interrupts cancer cell progression and metastasis by suppressing cancer cell proliferation and survival. | (8) |
| **Apoptosis** | | |
| Apoptosis-inducing factor mitochondria associated 1 | It triggers caspase-independent apoptosis. Induction of apoptosis results in the translocation of this protein to the nucleus, which affects chromosome condensation and fragmentation. | (9-11) |
| Cytochrome -c, -P450 | inhibit proliferation and prime apoptosis. Regulate IGF-1R/Akt/p53 Signaling Pathway | (12, 13) |
| Annexin-V, -A5, A7 | Protein with high affinity to upregulated and externally expressed phosphatidylserine (PS) on cancer cells. Annexin V can be used as a novel angiogenesis inhibitor in tumor therapy. | (14, 15) |
| Plasminogen activator inhibitors (PAI) | PAI-1 directly binds to caspases as a mechanism of PAI-1-mediated cellular apoptosis. | (16) |
| Programmed cell death 6-interacting protein (ALIX) | it binds to the product of the PDCD6 gene, a protein needed for apoptosis, in a calcium-dependent manner. | (17) |
| **EMT** | | |
| CD109 antigen TGF-beta-1-binding protein) | a glycosylphosphatidylinositol (GPI)-anchored protein and a novel TGF-β co-receptor that negatively regulates TGF-β signaling and suppresses cell migration. | (18) |
| Filamin-A | Inhibition of Akt Signaling, cancer suppressor, regulates focal adhesion disassembly and suppresses cancer cell migration and invasion. | (19, 20) |
| Septin-6, -7 | tumor-suppressor that has been reported to suppress glioma cell migration and invasion. | (21) |
| Sciellin | mediates mesenchymal-to-epithelial transition | (22) |
| Matrin 3 | suppressed in vitro tumorigenicity, promoted apoptotic cell death, and inhibited EMT, migration, and invasion in BLBC/TNBC cells. | (23) |
| Ras-related protein Rab-11A | regulating EGFR and EpCAM exosome secretion and negatively regulating migration and invasion of HNSCC cells. | (24) |
| Cytochrome B5 type A | alleviates cancer metastasis | (25) |
| Catenin alpha-1 (Alpha E-catenin) | α-catenin bound to the cadherin-β-catenin complex bridges these components to actin. It facilitates cell-cell adhesion which is responsible for tumor-suppressive action. | (26, 27) |
| **Angiogenesis** | | |
| Thrombospondin type-1, -2 | inhibit angiogenesis in TME. | (28) |
| Cytochrome b5 type B | attenuating angiogenesis | (25, 29) |
| Endostatin | inhibits endothelial proliferation and potently inhibits angiogenesis and tumor growth | (30) |
| Tissue inhibitors of metalloproteinase (TIMP) -1, -2, -3 and -4 | inhibit angiogenesis in TME. | (31, 32) |
| **Differentiation** | | |
| Macrophage-capping protein (Actin regulatory protein CAP-G) | Regulation of actin cytoskeleton during NB differentiation | (33, 34) |
| Neuroplastin | it is implicating in neuronal differentiation | (35) |
| Drebrin A | is an actin-binding protein that plays a vital role in regulating spine morphology and synaptic plasticity. | (36) |
| **Glycolysis** | | |
| Glutathione S-transferase P | Glucose homeostasis | (37) |
| Pyruvate kinase PKM | a limiting glycolytic enzyme | (38) |
| Citrate synthase | Inhibition of Glycolysis | (39) |
| ATP-dependent 6-phosphofructokinase | In the glycolytic cycle, ATP-dependent 6-phosphofructokinase (PFK) is a rate-limiting enzyme | (40) |
| **OPHOS** | | |
| ATP synthase subunit alpha | mitochondrial (ATP synthase F1 subunit alpha) | (41) |
| Succinate dehydrogenase | Oxidation of succinate and mitochondrial hyperpolarization drive ROS production | (42) |
| Paraoxonase 2 (PON2) | modulates ROS production and overexpression of PON2 effectively protecting mitochondria from antimycin- or oligomycin-mediated mitochondrial dysfunction. | (43) |
| Peroxiredoxin-1 | It is a ubiquitous family of redox-regulating proteins that are reported of potential to eliminate various reactive oxygen species (ROS) | (44) |

**References**

1. Hua YQ, Zhang K, Sheng J, Ning ZY, Li Y, Shi WD, et al. NUCB1 Suppresses Growth and Shows Additive Effects With Gemcitabine in Pancreatic Ductal Adenocarcinoma via the Unfolded Protein Response. Frontiers in cell and developmental biology. 2021;9:641836.

2. Leali D, Alessi P, Coltrini D, Ronca R, Corsini M, Nardo G, et al. Long pentraxin-3 inhibits FGF8b-dependent angiogenesis and growth of steroid hormone-regulated tumors. Molecular cancer therapeutics. 2011;10(9):1600-10.

3. Giacomini A, Matarazzo S, Pagano K, Ragona L, Rezzola S, Corsini M, et al. A long pentraxin-3-derived pentapeptide for the therapy of FGF8b-driven steroid hormone-regulated cancers. Oncotarget. 2015;6(15):13790-802.

4. Ronca R, Alessi P, Coltrini D, Di Salle E, Giacomini A, Leali D, et al. Long pentraxin-3 as an epithelial-stromal fibroblast growth factor-targeting inhibitor in prostate cancer. The Journal of pathology. 2013;230(2):228-38.

5. Devarajan A, Su F, Grijalva V, Yalamanchi M, Yalamanchi A, Gao F, et al. Paraoxonase 2 overexpression inhibits tumor development in a mouse model of ovarian cancer. Cell death & disease. 2018;9(3):392.

6. Menges CW, Altomare DA, Testa JR. FAS-associated factor 1 (FAF1): diverse functions and implications for oncogenesis. Cell cycle (Georgetown, Tex). 2009;8(16):2528-34.

7. Bu S, Zhang Q, Wang Q, Lai DJIJoO. Human amniotic epithelial cells inhibit growth of epithelial ovarian cancer cells via TGF‑β1-mediated cell cycle arrest. 2017;51(5):1405-14.

8. Murayama Y, Oritani K, Tsutsui SJWJoGW. Novel CD9-targeted therapies in gastric cancer. 2015;21(11):3206.

9. Wu M, Xu L-G, Li X, Zhai Z, Shu H-BJJoBC. AMID, an apoptosis-inducing factor-homologous mitochondrion-associated protein, induces caspase-independent apoptosis. 2002;277(28):25617-23.

10. Ji W, Zhang L, Ma C, Xu X, Li S, Xia H, et al. Newly synthesized AIFM1 determines the hypersensitivity of T lymphocytes to STING activation-induced cell apoptosis. 2023;42(4).

11. Qiu Y, Wang H, Fan M, Pan H, Guan J, Jiang Y, et al. Impaired AIF-CHCHD4 interaction and mitochondrial calcium overload contribute to auditory neuropathy spectrum disorder in patient-iPSC-derived neurons with AIFM1 variant. 2023;14(6):375.

12. Liu Z, Zhao X, Zhang L, Pei BJOl. Cytochrome C inhibits tumor growth and predicts favorable prognosis in clear cell renal cell carcinoma. 2019;18(6):6026-32.

13. Mo H-Y, Wei Q-Y, Zhong Q-H, Zhao X-Y, Guo D, Han J, et al. Cytochrome P450 27C1 level dictates lung cancer tumorigenicity and sensitivity towards multiple anticancer agents and its potential interplay with the IGF-1R/Akt/p53 signaling pathway. 2022;23(14):7853.

14. Kang TH, Park JH, Yang A, Park HJ, Lee SE, Kim YS, et al. Annexin A5 as an immune checkpoint inhibitor and tumor-homing molecule for cancer treatment. 2020;11(1):1137.

15. Zhang X, Huo L, Jin H, Han Y, Wang J, Zhang Y, et al. Anti-cancer activity of Annexin V in murine melanoma model by suppressing tumor angiogenesis. 2017;8(26):42602.

16. Balsara RD, Ploplis VAJT, haemostasis. Plasminogen activator inhibitor-1: the double-edged sword in apoptosis. 2008;100(12):1029-36.

17. Hashemi M, Yousefi J, Hashemi SM, Amininia S, Ebrahimi M, Taheri M, et al. Association between programmed cell death 6 interacting protein insertion/deletion polymorphism and the risk of breast cancer in a sample of Iranian Population. 2015;2015(1):854621.

18. Li C, Hancock MA, Sehgal P, Zhou S, Reinhardt DP, Philip AJBJ. Soluble CD109 binds TGF-β and antagonizes TGF-β signalling and responses. 2016;473(5):537-.

19. Xu Y, Bismar TA, Su J, Xu B, Kristiansen G, Varga Z, et al. Filamin A regulates focal adhesion disassembly and suppresses breast cancer cell migration and invasion. 2010;207(11):2421.

20. Campos LS, Rodriguez YI, Leopoldino AM, Hait NC, Bergami PL, Castro MG, et al. Filamin A expression negatively regulates sphingosine-1-phosphate-induced NF-κB activation in melanoma cells by inhibition of Akt signaling. 2016.

21. Jiang H, Hua D, Zhang J, Lan Q, Huang Q, Yoon J-G, et al. MicroRNA-127-3p promotes glioblastoma cell migration and invasion by targeting the tumor-suppressor gene SEPT7. 2014;31(5):2261-9.

22. Chou C-K, Fan C-C, Lin P-S, Liao P-Y, Tung J-C, Hsieh C-H, et al. Sciellin mediates mesenchymal-to-epithelial transition in colorectal cancer hepatic metastasis. 2016;7(18):25742.

23. Yang J, Lee SJ, Kwon Y, Ma L, Kim JJBr. Tumor suppressive function of Matrin 3 in the basal-like breast cancer. 2020;53.

24. Yoshida K, Htike K, Eguchi T, Kawai H, Eain HS, Tran MT, et al. Rab11 suppresses head and neck carcinoma by regulating EGFR and EpCAM exosome secretion. 2024;66(1):205-16.

25. Guo H, Liang S, Wang Y, Zhou S, Yin D, Zhang S, et al. Cytochrome B5 type A alleviates HCC metastasis via regulating STOML2 related autophagy and promoting sensitivity to ruxolitinib. 2022;13(7):623.

26. Qian ZR, Li CC, Yamasaki H, Mizusawa N, Yoshimoto K, Yamada S, et al. Role of E-cadherin, α-, β-, and γ-catenins, and p120 (cell adhesion molecules) in prolactinoma behavior. 2002;15(12):1357-65.

27. Drees F, Pokutta S, Yamada S, Nelson WJ, Weis WIJC. α-catenin is a molecular switch that binds E-cadherin-β-catenin and regulates actin-filament assembly. 2005;123(5):903-15.

28. Lawler PR, Lawler JJCSHpim. Molecular basis for the regulation of angiogenesis by thrombospondin-1 and-2. 2012;2(5):a006627.

29. Ming H, Lan Y, He F, Xiao X, Zhou X, Zhang Z, et al. Cytochrome b5 reductase 2 suppresses tumor formation in nasopharyngeal carcinoma by attenuating angiogenesis. 2015;34:1-9.

30. O'Reilly MS, Boehm T, Shing Y, Fukai N, Vasios G, Lane WS, et al. Endostatin: an endogenous inhibitor of angiogenesis and tumor growth. 1997;88(2):277-85.

31. Niknejad H, Yazdanpanah G, Ahmadiani AJC, research t. Induction of apoptosis, stimulation of cell-cycle arrest and inhibition of angiogenesis make human amnion-derived cells promising sources for cell therapy of cancer. 2016;363(3):599-608.

32. Johnson MD, Kim HRC, Chesler L, Tsao‐Wu G, Polverini PJ, Bouck NJJocp. Inhibition of angiogenesis by tissue inhibitor of metalloproteinase. 1994;160(1):194-202.

33. Edwards M, Zwolak A, Schafer DA, Sept D, Dominguez R, Cooper JAJNrMcb. Capping protein regulators fine-tune actin assembly dynamics. 2014;15(10):677-89.

34. Barth M, Toto Nienguesso A, Navarrete Santos A, Schmidt CJCB. Quantitative proteomics and in-cell cross-linking reveal cellular reorganisation during early neuronal differentiation of SH-SY5Y cells. 2022;5(1):551.

35. Owczarek S, Soroka V, Kiryushko D, Larsen MH, Yuan Q, Sandi C, et al. Neuroplastin‐65 and a mimetic peptide derived from its homophilic binding site modulate neuritogenesis and neuronal plasticity. 2011;117(6):984-94.

36. Takahashi H, Mizui T, Shirao TJJon. Down‐regulation of drebrin A expression suppresses synaptic targeting of NMDA receptors in developing hippocampal neurones. 2006;97:110-5.

37. Zhang J, Grek C, Ye Z-W, Manevich Y, Tew KD, Townsend DM. Pleiotropic functions of glutathione S-transferase P. Advances in cancer research. 122: Elsevier; 2014. p. 143-75.

38. Israelsen WJ, Vander Heiden MG, editors. Pyruvate kinase: Function, regulation and role in cancer. Seminars in cell & developmental biology; 2015: Elsevier.

39. Ren J-G, Seth P, Ye H, Guo K, Hanai J-i, Husain Z, et al. Citrate suppresses tumor growth in multiple models through inhibition of glycolysis, the tricarboxylic acid cycle and the IGF-1R pathway. 2017;7(1):4537.

40. Almacellas E, Pelletier J, Manzano A, Gentilella A, Ambrosio S, Mauvezin C, et al. Phosphofructokinases axis controls glucose-dependent mTORC1 activation driven by E2F1. 2019;20:434-48.

41. Jonckheere AI, Smeitink JA, Rodenburg RJJJoimd. Mitochondrial ATP synthase: architecture, function and pathology. 2012;35:211-25.

42. Mills EL, Kelly B, Logan A, Costa AS, Varma M, Bryant CE, et al. Succinate dehydrogenase supports metabolic repurposing of mitochondria to drive inflammatory macrophages. 2016;167(2):457-70. e13.

43. Devarajan A, Bourquard N, Hama S, Navab M, Grijalva VR, Morvardi S, et al. Paraoxonase 2 deficiency alters mitochondrial function and exacerbates the development of atherosclerosis. 2011;14(3):341-51.

44. Neumann CA, Krause DS, Carman CV, Das S, Dubey DP, Abraham JL, et al. Essential role for the peroxiredoxin Prdx1 in erythrocyte antioxidant defence and tumour suppression. 2003;424(6948):561-5.
