# Supplementary material for: Amniotic membrane promotes doxorubicin potency by suppressing SH-SY5Y neuroblastoma cell angiogenesis
Source: BMC Cancer. 2025 Jun 19;25:1021. doi: 10.1186/s12885-025-14442-z (PMC12180182; doi:10.1186/s12885-025-14442-z)
Supplement: Supplementary file 2 — Supplementary Material 2. [file 12885_2025_14442_MOESM2_ESM.docx]

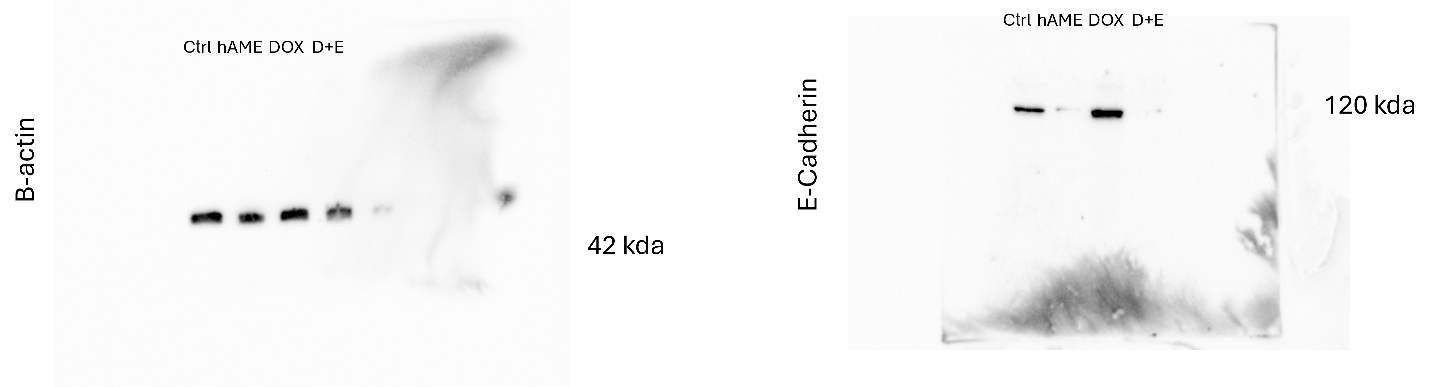


**Fig. 1 Full uncropped blot for b-actin and E-Cadherin.**


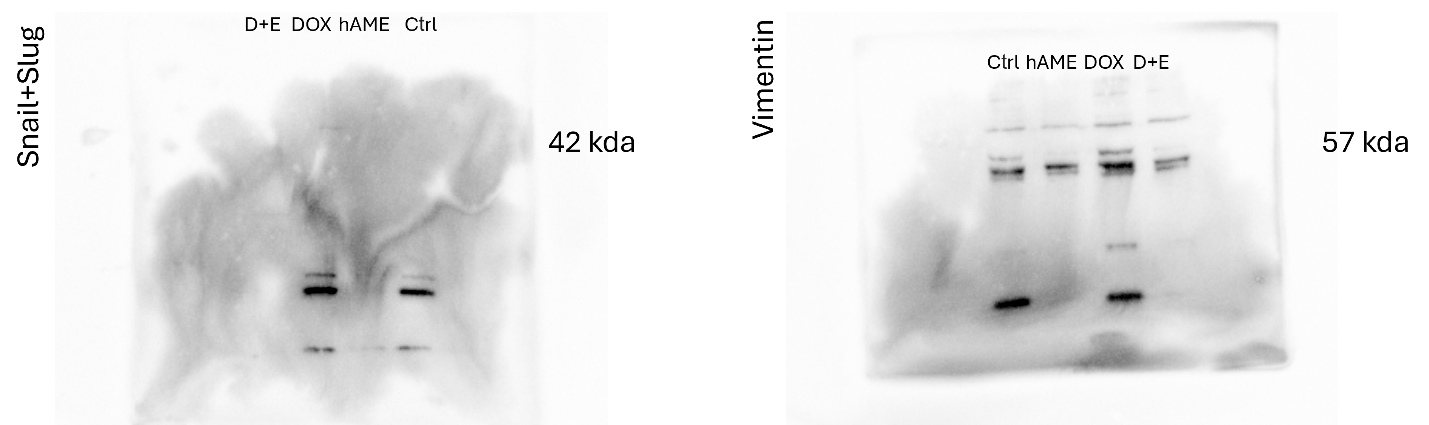


**Fig. 2 Full uncropped blot for Snail+Slug and Vimentin.**


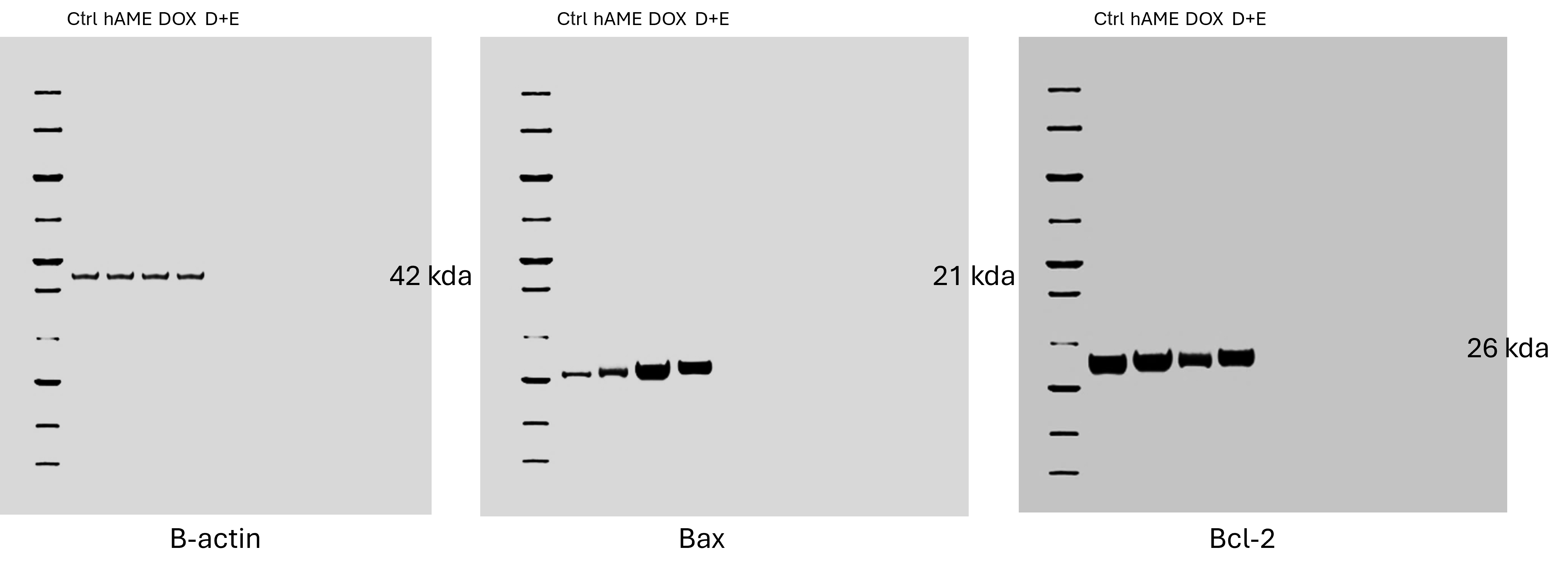


**Fig. 3 Full uncropped blot for BAX, Bcl-2, and β-actin.**

**
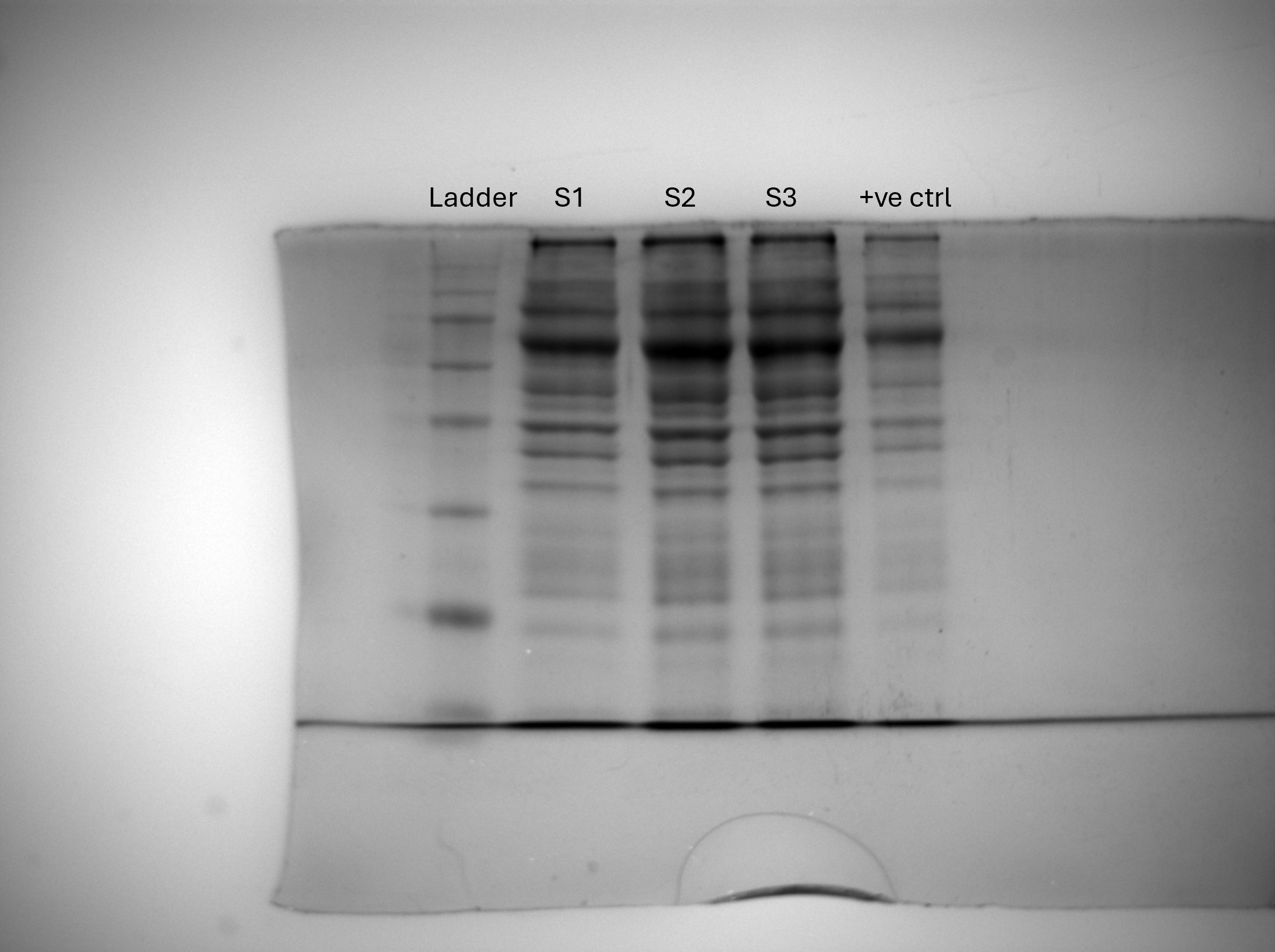
**

**Fig. 4: Full uncropped gel for supplementary file 1, Fig. 1D.**

### Table. 1: The primers and their sequence used in the qRT-PCR experiment.

| **Gene (protein)** | **Primer** | **Sequence (5' to 3')** |
| --- | --- | --- |
| **House-keeping Gene** | | |
| β-actin | Forward | CACCATTGGCAATGAGCGGTTC |
|  | Reverse | AGGTCTTTGCGGATGTCCACGT |
| **Glycolytic Markers** | | |
| TPI1 | Forward | CGAGCAGACAAAGGTCATCGCA |
|  | Reverse | TCGGAGCTTCTCGTGTACTTCC |
| ALDOC | Forward | CATTCTGGCTGCGGATGAGTCT |
|  | Reverse | CACACGGTCATCAGCACTGAAC |
| GLUT1 | Forward | TTGCAGGCTTCTCCAACTGGAC |
|  | Reverse | CAGAACCAGGAGCACAGTGAAG |
| HK2 | Forward | GAGTTTGACCTGGATGTGGTTGC |
|  | Reverse | CCTCCATGTAGCAGGCATTGCT |
| **OXPHOS Markers** | | |
| NDUFA5 | Forward | TAGAAGACCAACTTCAAGGCGGT |
|  | Reverse | AGGAGGCTCTTCCACTAATGGC |
| NDUFA10 | Forward | TGGCTCAAGCAGGACAATCGCA |
|  | Reverse | AGACACGGTCAGTCTGATGAGC |
| COX11 | Forward | GAACAAGACGACCCTCACTTACG |
|  | Reverse | GCAACTGCTGATCCTCCAAGTC |
| ATP6V1H | Forward | CGGGTCAATGAGTACCGCTTTG |
|  | Reverse | GATACTGGAGCTGAAAGCCACAC |
| SPI1 | Forward | GCGACCATTACTGGGACTTCC |
|  | Reverse | GGGTATCGAGGACGTGCAT |
| **Proliferation Markers** | | |
| P21 | Forward | GATGGCACCAGAGGTGGTTA |
|  | Reverse | TCCCGAAATATTGGGGAAAG |
| hTERT | Forward | CGGAAGAGTGTCTGGAGCAA |
|  | Reverse | GGATGAAGCGGAGTCTGGA |
| TOP1 | Forward | GGCACTGGTATCCTGAAAAGCC |
|  | Reverse | GTGGCAGATTGCCACGAACAT |
| c-MYC | Forward | GCGACTCTGAGGAGGAA |
|  | Reverse | TGCGTAGTTGTGCTGATG |
| K-Ras | Forward | TGTTCACAAAGGTTTTGTCTCC |
|  | Reverse | CCTTATAATAGTTTCCATTGCCTTG |
| CCNA1 | Forward | GCACACTCAAGTCAGACCTGCA |
|  | Reverse | ATCACATCTGTGCCAAGACTGGA |
| N-MYC | Forward | CGCAAAAGCCACCTCTCATTA |
|  | Reverse | TCCAGCAGATGCCACATAAGG |
| PHOX2B | Forward | TCAGGGACCACCAGAGCAGT |
|  | Reverse | GGTGAAAGTGGTGCGGATGC |
| **Apoptotic Markers** | | |
| BAX | Forward | CAAACTGGTGCTCAAGGCCC |
|  | Reverse | GAGACAGGGACATCAGTCGC |
| BCL-2 | Forward | GGATAACGGAGGCTGGGATG |
|  | Reverse | TGACTTCACTTGTGGCCCAG |
| Caspase-3 | Forward | ACAGTGGAACTGACGATGATATG |
|  | Reverse | TCCCTTGAATTTCTCCAGGAATAG |
| P53 | Forward | GGAAGAGAATCTCCGCAAGAA |
|  | Reverse | AGCTCTCGGAACATCTCGAAG |
| **Neuronal Differentiation Associated Markers** | | |
| TuJ1 | Forward | TCCAGGAGCTGTTCAAGCG |
|  | Reverse | TCGGACACCAGGTCGTTC |
| NCAM | Forward | AACAAAGCATGATGGGTGAA |
|  | Reverse | GTCTGTGGTGTTGGAAATGC |
| MAP2 | Forward | GGGCCTTTTCTTTGAAATCTAGTTT |
|  | Reverse | CAAATGTGGCTCTCTGAAGAACA |
| ID 1 | Forward | GCTGTTACTCACGCCTCAA |
|  | Reverse | CAACTGAAGGTCCCTGATGTAG |
| ID2 | Forward | CAAGAAGGTGAGCAAGATGGA |
|  | Reverse | GGTGATGCAGGCTGACAATA |
| ID3 | Forward | CGACATGAACCACTGCTACTC |
|  | Reverse | GATGACGCGCTGTAGGATTT |
| KLF4 | Forward | AGAGGAGCCCAAGCCAAAG |
|  | Reverse | CGTCCCAGTCACAGTGGTAAG |
| **Angiogenesis Markers** | | |
| VEGF | Forward | ATCTGCATGGTGATGTTGGA |
|  | Reverse | GGGCAGAATCATCACGAAGT |
| PDGF | Forward | GATACTTTGCGCGCACACAC |
|  | Reverse | GGTTTTCTCTTTGCAGCGAGG |
| MMP-3 | Forward | CACTCACAGACCTGACTCGGTT |
|  | Reverse | AAGCAGGATCACAGTTGGCTGG |
| MMP13 | Forward | TTCGGCTTAGAGGTGACTGGC |
|  | Reverse | TTCACCCACATCAGGAACCCC |
| **MET Markers** | | |
| SLUG | Forward | TGTTGCAAGTGAGGGCAAGAA |
|  | Reverse | GACCCTGGTTGCTTCAAGGA |
| TWIST-1 | Forward | CGGGAGTCCGCAGTCTTA |
|  | Reverse | GCTTGAGGGTCTGAATCTTG |
| ZEB1 | Forward | GATGATGAATGCGAGTCAGATGC |
|  | Reverse | CTGGTCCTCTTCAGGTGCC |
| VIMENTIN | Forward | TGTCCAAATCGATGTGGATGTTTC |
|  | Reverse | TTGTACCATTCTTCTGCCTCCTG |
